# Toward simulating offshore oilfield conditions: insights into microbiologically influenced corrosion from a dual anaerobic biofilm reactor

**DOI:** 10.1128/aem.02221-24

**Published:** 2025-03-04

**Authors:** Liam M. Jones, Niall Hanrahan, Maria Salta, Torben Lund Skovhus, Kathryn Thomas, Timothy Illson, Julian Wharton, Jeremy S. Webb

**Affiliations:** 1 School of Biological Sciences, University of Southampton 7423 , Southampton, United Kingdom; 2 School of Chemistry, Faculty of Engineering and Physical Sciences, University of Southampton 7423, Southampton, United Kingdom; 3 Institute for Life Sciences, University of Southampton 7423, Southampton, United Kingdom; 4 MIC and Biofilm Department, Endures 486628 , Bevesierweg, Den Helder, Netherlands; 5 Research Centre for Built Environment, Climate and Water Technology, VIA University College, Horsens, Denmark; 6 DNV, Loughborough, United Kingdom; 7 School of Engineering, University of Southampton 7423 , Southampton, United Kingdom; 8 National Biofilms Innovation Centre 631829 , Southampton, United Kingdom; Michigan State University, East Lansing, Michigan, USA

**Keywords:** biofilms, microbiologically influenced corrosion, electroactive microorganisms, standardization, carbon steel, multiple lines of evidence

## Abstract

Oilfield systems are a multifaceted ecological niche, which consistently experiences microbiologically influenced corrosion. However, simulating the environmental conditions of an offshore system within the laboratory is notoriously difficult. A novel dual anaerobic biofilm reactor protocol allowed a complex mixed-species marine biofilm to be studied. Interestingly, electroactive corrosive bacteria and fermentative electroactive bacteria growth was supported within the biofilm microenvironment. Critically, the biotic condition exhibited pits with a greater average area, which is characteristic of microbiologically influenced corrosion. This research seeks to bridge the gap between experimental and real-world scenarios, ultimately enhancing the reliability of biofilm management strategies in the industry.

**IMPORTANCE:**

It is becoming more widely understood that any investigation of microbiologically influenced corrosion requires a multidisciplinary focus on multiple lines of evidence. Although there are numerous standards available to guide specific types of testing, there are none that focus on integrating biofilm testing. By developing a novel dual anaerobic reactor model to study biofilms, insights into the different abiotic and biotic corrosion mechanisms under relevant environmental conditions can be gained. Using multiple lines of evidence to gain a holistic understanding, more sustainable prevention and mitigation strategies can be designed. To our knowledge, this is the first time all these metrics have been combined in one unified approach. The overall aim of this paper was to explore recent advances in biofilm testing and corrosion research and provide recommendations for future standards being drafted. However, it is important to note that this article itself is not intending to serve as a standard.

## INTRODUCTION

The formation and behavior of biofilm communities in oil and gas systems, particularly those involving carbon steel (CS) surfaces in contact with produced water (PW), is a critical area of study due to its implications for material and infrastructure integrity. The presence of certain types of microorganisms can contribute to infrastructure complications, such as corrosion, souring, and biofouling (1, 2). Although some metal loss is expected and accounted for during the design of the infrastructure, higher rates of corrosion, unless detected and mitigated early, can necessitate costly repairs or replacements (3). PW is one of the most common sampling sources within oilfield systems, which is typically nutrient-rich, due to a high organic content. Microbial diversity and biomass can vary greatly but are often characterized to be low in oilfield systems due to extreme conditions and chemical treatments (4–6). Moreover, environmental samples are notoriously difficult to culture using standard laboratory techniques. This is because PW typically contains a diverse and complex mixture of organic compounds, including hydrocarbons and organic acids. However, PW does not contain rich carbon sources, including amino acids, peptides, and sugars. Thus, simulating the environmental conditions of an offshore system within the laboratory while supporting the growth of environmental cultures can be challenging. Consequently, laboratory studies on biofilms have typically been conducted under simplified conditions. However, there is a need to replicate the complex environmental parameters of offshore systems more closely to ensure the relevance of findings and any mitigation strategies employed.

Microbiologically influenced corrosion (MIC) is a complex and widespread phenomenon in which microbial activities, often involving biofilms, accelerate the deterioration of metals and other materials in diverse environments, leading to significant economic, structural, and operational challenges in industries such as oil and gas, water treatment, and maritime operations (7). MIC is notoriously difficult to detect and monitor within industry. Despite the advent of molecular tools and improved microbial monitoring strategies for oil and gas operations, specific underlying MIC mechanisms in pipelines remain largely enigmatic (3). Consequently, MIC is a particularly difficult corrosion mechanism to manage. Oilfield systems contain multiphase fluids, including crude oil, gas, as well as PW (8). This multifaceted ecological niche consists of many undetermined thermophilic and mesophilic archaea/bacteria (8). These uncharacterized microorganisms may metabolize organic and inorganic compounds in crude oil and metal pipelines under extreme environmental conditions (9, 10). Moreover, these microbial communities have the potential to change the redox chemistry within oilfield systems (11, 12). Furthermore, PW may also contain a high concentration of minerals, such as ${Cl}^{-}$ and ${SO}_{4}^{2-}$, which may influence the community dynamics within the biofilm. This will impact initial biofilm formation and influence the predominant microbial metabolisms present. Therefore, any MIC processes at the interface of the biofilm and the metal surface will be affected (12–14).

The most common corrosion mechanisms reported in oilfield systems are linked to $H_{2}S$ and/or ${CO}_{2}$ acid gases. These gases readily dissolve into PW, resulting in aqueous corrosive environments (3). However, these are abiotic corrosion mechanisms. Abiotic corrosion mechanisms, such as acid gas corrosion, can be modeled *in silico* (15), or alternatively, they can be simulated in laboratory reactors (16) to understand the rates of metal loss expected under specific field conditions. This is critical for industry, as field conditions such as flow rate, temperature, water chemistry, and partial pressure of acid gases will all impact reported corrosion rates (*CR*s) (3). Additionally, laboratory testing of abiotic corrosion mechanisms provides a robust approach to testing the efficacy of different mitigation strategies. Conversely, biotic corrosion mechanisms have not been adequately modeled within the laboratory for mixed-species biofilms from an industrial perspective. Similarly, the efficacy of biocides, applied to control MIC, has also not been adequately modeled. Thus, the development of such a model protocol is important for the effective management of MIC mechanisms. Currently, field-based detection and monitoring is primarily employed for the effective management of MIC corrosion mechanisms (3). Microbiological assessment is routinely performed to detect the presence of MIC causative microorganisms and evaluate the effectiveness of biocide treatments used to mitigate against MIC (17). However, by the time MIC is detected and diagnosed as the root cause, it may already be too late to effectively mitigate due to the recalcitrant nature of biofilms.

It is well documented that sessile microorganisms within biofilms attach to metal surfaces, which leads to electrochemical MIC processes. It is widely accepted that there are distinct phylogenetic groups such as iron-reducing bacteria (IRB), sulfate-reducing bacteria (SRB), and acid-producing bacteria (APB), which actively participate in MIC (8, 18–20). Moreover, it is becoming increasingly recognized that electroactive microorganisms, capable of exchanging electrons with their extracellular environment, play a key role in extracellular electron transfer (EET) and MIC (21). However, long-term studies on the influence of mixed-species biofilms on MIC in oilfield systems are limited. Most laboratory studies on MIC are typically short to medium term, often focusing on specific aspects of the corrosion process, such as biofilm formation, *CR*, or the identification of corrosive microbial species. Although these studies provide valuable insights, they may not fully capture the long-term dynamics and cumulative effects of MIC, particularly under conditions that closely mimic real-world environments. Recent studies from Elumalai et al. found that crude oil reservoirs were dominated by Proteobacteria, Actinobacteria, and Firmicutes classes (8, 22). Proteobacteria biofilms have been associated with various types of corrosion, with *Pseudomonas* sp. being shown to form a thin biofilm with corrosion deposits and causing the reduction of ${Fe}^{3+}$ to ${Fe}^{2+}$ on the metal surface (23, 24). Moreover, Proteobacteria species have been reported to consume hydrocarbons as a nutrient source and have the proficiency to degrade aromatic hydrocarbons for their metabolic pathways (25).

Long-term studies are challenging to conduct due to the time, resources, and complexities involved in replicating the dynamic and multifaceted conditions found in oil and gas systems. However, the need for such studies is increasingly recognized, as they can provide more comprehensive data on how biofilms evolve over time, how microbial communities interact with materials, and how these interactions contribute to long-term corrosion processes. Understanding these factors is crucial for developing effective mitigation strategies to protect infrastructure in the oil and gas industry.

This study investigates the impact of simulated environmental conditions on natural mixed-species biofilm communities, aiming to provide deeper insights into their development, community dynamics, and potential to induce MIC within a novel dual bioreactor protocol that simulates offshore oil and gas environments. It is important to note that shear stress, pressure, acid gas corrosion, and the presence of crude oil are all important considerations that are not simulated here. This study builds off a previous experiment (7) and further aims to establish a reproducible bioreactor-based model for MIC research. By aligning laboratory conditions with those encountered *in situ*, this research seeks to bridge the gap between experimental and real-world scenarios, ultimately enhancing the reliability of biofilm management strategies in the industry. Importantly, utilizing multiple lines of evidence (MLOE) (26), the protocol incorporates a multi-disciplinary approach to gain a holistic understanding of biofilms and MIC. It was hypothesized that over time, the biotic condition would exhibit a greater incidence of pitting compared with the abiotic condition.

## MATERIALS AND METHODS

### Test conditions

Two anaerobic CDC (Centers for Disease Control) biofilm reactors (Biosurface Technologies Corporation) were used: an abiotic control reactor and a biotic test reactor (key dimensions: 22 cm reactor height and 12 cm internal diameter; 21 cm coupon holder rod; 1.27 cm coupon diameter), along with Masterflex High-Performance Precision Pump Tubing, C-Flex (Model 06424), which is a Thermoplastic Elastomer (TPE) with low gas permeability. Sterile carbon steel coupons were fixed in reactors and exposed to two separate conditions for 28 days. Anaerobic conditions were maintained throughout the test by initially sparging the system with nitrogen gas (oxygen-free nitrogen) (BOC Nitrogen [Oxygen Free], 44 W) over an initial 3-day batch phase. For this experiment, only nitrogen gas, and not a mixture of nitrogen/carbon dioxide, was used for strict oxygen removal, and to support the growth of a broad range of anaerobic microorganisms. Anaerobic conditions, considered to be hypoxic or low $O_{2}$, are characterized as a system with low concentrations ranging between 1% and 30% saturation. Strict obligate anaerobes will not survive if there is more than half a percent $O_{2}$ in the environment, whereas moderate obligate anaerobes can still grow in a 2%–8% $O_{2}$ environment (27). PW collected from an offshore platform (first stage separator: 50°C) was used as the test medium. The bulk-produced water, used in the reactors, was prepared by filter sterilisation prior to use to ensure sterility, using a 0.2 µm Vivaflow TFF Cassette, PES (Sartorius). The test media composition can be seen in Table S1 . Resazurin was added as a redox indicator, as it is colorless under oxygen-free conditions but changes to pink color in an oxygen-containing environment. Agitation of the reactor baffles was set to 50 rpm to maintain a homogeneous solution. The reactor temperature was set to 40°C to better simulate the environmental condition of the offshore system. Prior to inoculating the biotic reactor, a 3-day pre-culture was prepared in a blue-cap flask (50 mL), consisting of 10% marine sediment with the remainder filter-sterilized PW media. The pre-culture PW was prepared by adding 1 g/L of the yeast extract to filtered produced water and autoclaving it. It was acknowledged, for the pre-culture, that yeast extract contains redox mediators that may adsorb onto the electrode surfaces and chelate metal ions, and the test matrix was designed to highlight any significant interference (28). The biotic reactor was inoculated using a sterile syringe, where 10% of the working reactor volume (35 mL) was added as the inoculum. Initial adenosine triphosphate (ATP) measurements were taken from the pre-culture, and long-term frozen stocks were prepared using 20% glycerol. Fig. S2a shows the full experimental setup, with Fig. S2b illustrating the three-electrode cell setup within each anaerobic CDC biofilm reactor. Both reactors were operated in batch mode for the first 3 days to allow settlement and facilitate biofilm formation in the biotic reactor. After this period, the reactors were switched to the continuous flow of fresh media at a rate of 0.2 mL min^–1^, which replaced about 50% of the 600 mL total volume daily (288 mL day^−1^).

### Microbial consortia

The sheltered zone littoral sediment microbial consortia were collected at a depth between 10 and 15 cm below the sediment surface during low tide from Langstone Harbour, United Kingdom (50°50'11.9"N 0°58'47.5"W). The coastal/estuarine marine sediment (very fine and cohesive mud and silt deposits) was selected to sample microorganisms living under low oxygen conditions. The sediment samples were added to 500 mL of the PW medium and stored at 37°C in an anaerobic chamber to maximize the recovery of the diverse microbial populations. Mesophilic bacteria can survive and grow in temperatures between 10°C and 50°C (29). Thus, a tropic strategy to promote cell growth and viability was employed to maximize microbial recovery. The anaerobic chamber gas mixture consisted of 85% N_2_, 10% CO_2_, and 5% H_2_ (BOC Anaerobic Growth Mix, 290563 L). Long-term storage of sediment samples and microbial consortia was employed to create frozen stocks at –80°C.

### Carbon steel coupon preparation

UNS G10180 (AISI 1018) carbon steel disc coupons (Biosurface Technologies – RD128 CS), with dimensions of 12.7 mm diameter × 3.8 mm thickness were used as-received (AR) (*R*_a_ = 1.31906 ± .33859). The surface profiles and weights for all coupon samples were assessed prior to starting the experiment, on day 0, for surface profilometry and gravimetric analysis to be performed at the completion of the experiment after day 28. Three-dimensional (3D) surface profiles were taken using a 3D optical profilometer (Alicona imaging infinite focus microscope IFM G4 3.5). A Mettler AT201 was used to take five measurements of the initial weights of all coupons.

### Experimental setup

Before autoclaving, the two anaerobic CDC biofilm reactors were cleaned with detergent and allowed to dry. The empty reactors with attached tubing were placed in autoclavable bags; all tube openings and air filters (Millex, 0.2 µm) were covered in aluminium foil, with tube openings clamped shut. The rods, without coupons, were also covered in aluminum foil and placed in an autoclavable bag. The empty assembled reactors were autoclaved for 15 min at 121°C. After cooling, the reactors were transferred into a sterilized microbiological safety cabinet, along with all rods, carbon steel test coupons, as well as any sensors and electrodes. Working electrode rods were prepared in advance. For each working electrode rod, wires were soldered to each coupon separately. The coupon face with the soldered wire was then covered with a lacquer solution (Polishing Shop, Type 45 Stop Off Lacquer) and allowed to dry. To assemble the reactors, all rods with coupons were submerged in 99% ethanol for at least 10 s and then inserted into the autoclaved reactors. This was to disinfect the carbon steel coupons. Any sensors or electrodes used in place of a rod were also inserted, after also being sterilized with 99% ethanol for at least 10 s. The medium bottles and all tubing were connected to a microbiological safety cabinet. Once both reactors were fully assembled, they were transferred to the working area, with access to a N_2_ gas supply. The tubing was evenly split into each reactor to equalize the pressure gradient caused by the peristaltic pump (Matson Marlow 300 series).

### Sulfide analysis

Sulfide concentrations were monitored daily in each reactor using a Unisense, SULF-50 sulphide microsensor (50 µm diameter) and amplifier (Unisense, H_2_S UNIAMP). The microsensor measures the partial pressure of H_2_S gas, and the total concentration is a function of pH and temperature. The microsensor limit of detection is 0.3 µM, with a range from 0 to 300 µM sulfide in water. Calibration utilized the H_2_S and SULF sensor calibration kit (Unisense, CALKIT-H_2_S). Due to the nature of the experimental setup, it was not possible to calibrate the microsensors during the experiment. However, calibrations were performed both prior to starting the experiment and once the experiment had finished to confirm that the sensors were still calibrated. The SensorTrace Suite software application was used to collect the sulfide microsensor data. The sensor has a higher signal for zero right after it has been connected to the amplifier; thus, each microsensor collected readings for 5 min (approximately 300 data points) each day. This was to allow the sensor to stabilize.

### Surface profilometry and visual inspection

Corrosion products and biofilms were removed from the surface using the cleaning protocol as described previously for the gravimetric analysis. Three-dimensional profiling of the carbon steel surfaces was reconstructed using an Alicona imaging infinite focus microscope IFM G4 3.5. The images allowed the assessment of changes in surface roughness compared with the surface profiles obtained prior to testing. Additionally, ImageJ/Fiji was used for the quantitative determination of pit depth, width, height, percentage area, and to assess pit rate (*PR*) and pit density (*PD*). This analysis was performed on 30 total locations on six AR coupons (five locations each). The method involved applying a color threshold to depths greater than 5 µm. Then, the images were converted to a binary mask. Next, measurement parameters were selected for areas greater than 650 µm^2^. Finally, the images were analyzed to display counts, area, and average size of pits. The pit parameters were adapted from ASTM G48-11 (30). For pit rate analysis, the deepest pits from each image were captured using the Alicona. Pit rates were calculated using the formula described in NACE SP0775-2023 (31).

### Gravimetric analysis

Corrosion products and biofilms were removed following the ASTM G1-03 standard with a 15% inhibited hydrochloric acid described in NACE SP0775-2023 (31, 32). A stock solution was made of 37.5% HCl (Merck, Suprapur, 1.00318.0500) to which 10 g/L of 1,3-di-n-butyl-2 thiourea (DBT) (Merck, 8.20423.0250) was added. Immediately prior to use, the stock solution was diluted by slowly adding a measured volume of stock solution to an equal volume of deionized water with stirring. A Mettler AT201 was used to take five measurements of all coupons. Corrosion rates were determined by the gravimetric technique that considers the weight loss and surface area of the metal samples described in NACE SP0775-2023 (31).

### Electrochemical analysis

Electrochemical measurements were performed using a Gamry Instruments potentiostat (Ref 600 Plus). The electrochemical behaviors of the carbon steel coupons were evaluated using a three-electrode system consisting of a UNS G10180 coupon as the working electrode, graphite rod (Alfa Aesar, 99.9995%, 6.15 mm diameter, 152 mm long) as the counter electrode, and a silver/silver chloride (Ag/AgCl, 3.5 M KCl) reference electrode (Sentek, (AgCl) Double junction Reference Electrode). On day 1, after the test reactor was inoculated, both reactors were left for at least 1 h prior to performing any electrochemical measurements. Open-circuit potentials (OCP) were recorded for each coupon on day 1 prior to measuring linear polarization resistance (LPR) and electrochemical impedance spectroscopy (EIS). LPR and EIS were measured daily for each sample. LPR measurements were performed from ±10 mV with respect to *E*_OCP_ using a scan rate of 0.167 mV s^−1^. EIS measurements were performed at OCP with an applied 10 mV_rms_ sinusoidal potential signal with a frequency range of 10^−2^ to 10^5^ Hz. Potentiodynamic polarization measurements were performed at the end of the experiment on day 28 for each coupon from –0.200 mV to +0.200 V using the scan rate of 0.5 mV s^−1^. Standard procedures were followed when selecting an equivalent circuit best-fit using the Gamry Echem Analyst software: (i) the chi-squared (*χ*^2^) error was suitably minimized (*χ*^2^ ≤ 10^–4^) and (ii) the errors associated with each element were ranged between 0% and 5%.

### Confocal laser scanning microscopy and post-image analysis

The distribution of live and dead cells within biofilms was studied using confocal laser scanning microscopy (CLSM). Coupons were gently rinsed with sterile anaerobic PBS, with the following composition: NaCl 8 g, KCl 0.2 g, Na_2_HPO_4_ 1.44 g, KH_2_PO_4_ 0.245 g, and deionized water 1 L and stained using the FilmTracer Live/Dead biofilm viability kit (Invitrogen) according to the manufacturer’s instructions. Before imaging with a Leica SP8 confocal microscope, coupons were rinsed with sterile deionized water to remove the excess dyes and fixed using mowiol. Mowiol had the following composition: 2.4 g Mowiol, 6 mL deionized water, 12 mL 0.2 M Tris (pH 8.5), 0.01 g sodium azide, and 6 g glycerol. Images were obtained with a 63× magnification and glycerol immersion. The dyes used stained live cells with a green-fluorescent color (SYTO 9) and dead cells with a red color (propidium iodide). The *z*-stacked images were analyzed using Imaris software (Oxford Instruments).

### Microbial community analysis

After 28 days, six AR coupons were gently rinsed with PBS and then placed in a Falcon tube containing 10 mL of PW solution. Long-term frozen stocks were prepared using 20% glycerol for the bulk fluid, AR biofilm, and P biofilm samples from the biotic reactor. The sediment, 3-day pre-culture, day 28 bulk fluid, and AR biofilm frozen stocks were sent in triplicate for DNA extraction and 16S rRNA amplicon sequencing. PCR amplification was completed with primer pairs 347F and 800R. Library preparation and sequencing were performed for the V3 and V4 regions of the 16S rRNA gene targeting both bacteria and archaea. The microbiome analysis pipeline along with DNA extraction was performed by Eurofins Genomics LLC. The Illumina platform was used, sequencing on MiSeq with the 2 × 300 bp paired-end read module. Additionally, 2,293,909 raw reads were obtained. Taxonomic classification was performed using Kraken2 (v 2.1.1). Bioinformatics and data analysis were performed using Qiime2 (version 2023.5) software. Reference library k2_standard_08 gb_20240112 was used for taxonomic classification. To visualize the multivariate dispersion of the community composition, a principal component analysis (PCA) analysis was conducted employing GraphPad (version 10.0.2).

### ATP assay

The ATP concentration in both the abiotic and biotic reactors was determined by luminescence after reaction with luciferin-luciferase using the BacTiter-Glo Microbial Cell Viability Assay kit (Promega). The assay provides a method for determining the number of viable microbial cells in culture based on quantitation of the ATP present. ATP is the energy source of all living cells and is involved in many vital biochemical reactions. When cells die, they stop synthesizing ATP, and the existing ATP pool is quickly degraded. Higher ATP concentration indicates a higher number of living cells. All assays were performed according to the manufacturer’s instructions. Six AR coupons were gently rinsed with PBS and then immersed in a Falcon tube containing 10 mL of PW solution. Any cells were detached from the metal coupons using a cell scraper (Biologix). Both planktonic and sessile samples were processed with the BacTiter-Glo Microbial Cell Viability Assay kit, which measures ATP from as few as 10 microbial cells. The ATP concentrations were determined by measuring luminescence with a Clariostar Plus Multimode Microplate Reader (BMG Labtech). Planktonic cells in each reactor were determined following the same method described before; in this case, 10 mL of the bulk test solution was processed with the BacTiter-Glo Microbial Cell Viability Assay kit. Negative controls of PBS, deionized water, and PW solution were used to indicate no ATP activity.

### Corrosion product analysis

Analysis of the corrosion products and biofilms was performed by SEM-EDS and Raman microspectroscopy. Upon retrieval, the coupons were preserved under anaerobic conditions in an AnaeroBox with an AnaeroPack (ThermoFisher). For SEM, all images and Energy dispersive X-ray spectroscopy (EDS) measurements were taken using a Hitachi S-3400N II SEM and attached energy-dispersive X-ray spectrometer (Oxford Instruments). Imaging was performed at approximately 15 kV with a working distance of 10 mm at various magnifications (71×, 1,000×, and 3,000×). EDS analysis used the same parameters and magnifications. For each sample, the analyzed areas were scanned at randomly distributed areas over the sample surface (*n* = 15). EDS data were analyzed using AZTEC software before being compiled in Microsoft Excel. Raman microspectroscopy experiments were conducted using a Renishaw InVia Raman microscope (Renishaw, UK), with a Leica DM 2500 M bright field microscope and an automated 100 nm-encoded XYZ stage. The samples were excited using a 532 nm laser directed through a Nikon 50× long working distance air objective (NA = 0.5). Raman-scattered signals were separated from the laser illumination at 532 nm using a Rayleigh edge filter, and a diffraction grating (532 nm: 1,800 L/mm) dispersed the Raman-scattered light onto a Peltier-cooled CCD (1024 pixels × 256 pixels). Calibration of the Raman shift was carried out using an internal silicon wafer using a peak of 520 cm^−1^. Spectra were acquired over two or three accumulations of between 5 and 20 s each, using laser power of up to 3 mW. Spectra were acquired from a selection of points manually determined using the brightfield imaging mode of the microscope. The spectra obtained were processed using MATLAB (MathWorks).

## RESULTS

### Visual observations

Over the initial 3-day batch phase, the abiotic media was pink in appearance, and the coupons maintained the silver-gray metallic lustre of CS. After the first week, the abiotic media was beginning to become cloudier, but it was not until day 11 that the abiotic PW became orange-pink in coloration. Similarly, over the initial 3-day batch phase, the biotic coupons maintained the silver-gray appearance of the CS. However, the PW quickly became dark green/black in coloration on day 3. After the flow of fresh PW media was started on day 4, the bulk fluid became pink in appearance. Similarly, it was not until day 11 that the biotic PW became orange-pink in coloration. Upon dismantling of the reactors on day 28, and retrieval of the coupon rods, there was a significant difference in the coupon appearances, see Fig. S3. Additionally, the appearance of the waste from the different reactor conditions was significantly different. The abiotic waste media was orange-pink in coloration with reddish-brown granular deposits, whereas the biotic waste media was dark green/black in coloration. The abiotic surfaces had the presence of a black corrosion product across the entire coupon surfaces, with some reddish-brown granular deposits. However, the biotic surfaces were only partially covered by a black corrosion product, with the presence of a heterogeneous dark green/black biofilm. The silver-gray appearance of the CS was also partially visible upon retrieval.

### Sulfide analysis

All values were detected as below zero; as such, it was assumed that no $H_{2}S$ was present throughout the experiment. Alternatively, the PW poisoned the microsensors. Nonetheless, both conditions experienced similar readings. Unfortunately, *DO* concentrations could not be measured as the probe was not working. However, the pH was measured on day 28 for the media containers, waste containers, and reactor systems. All values recorded were between 7.01 and 7.47.

### Carbon steel surface analysis

**Fig 1:**
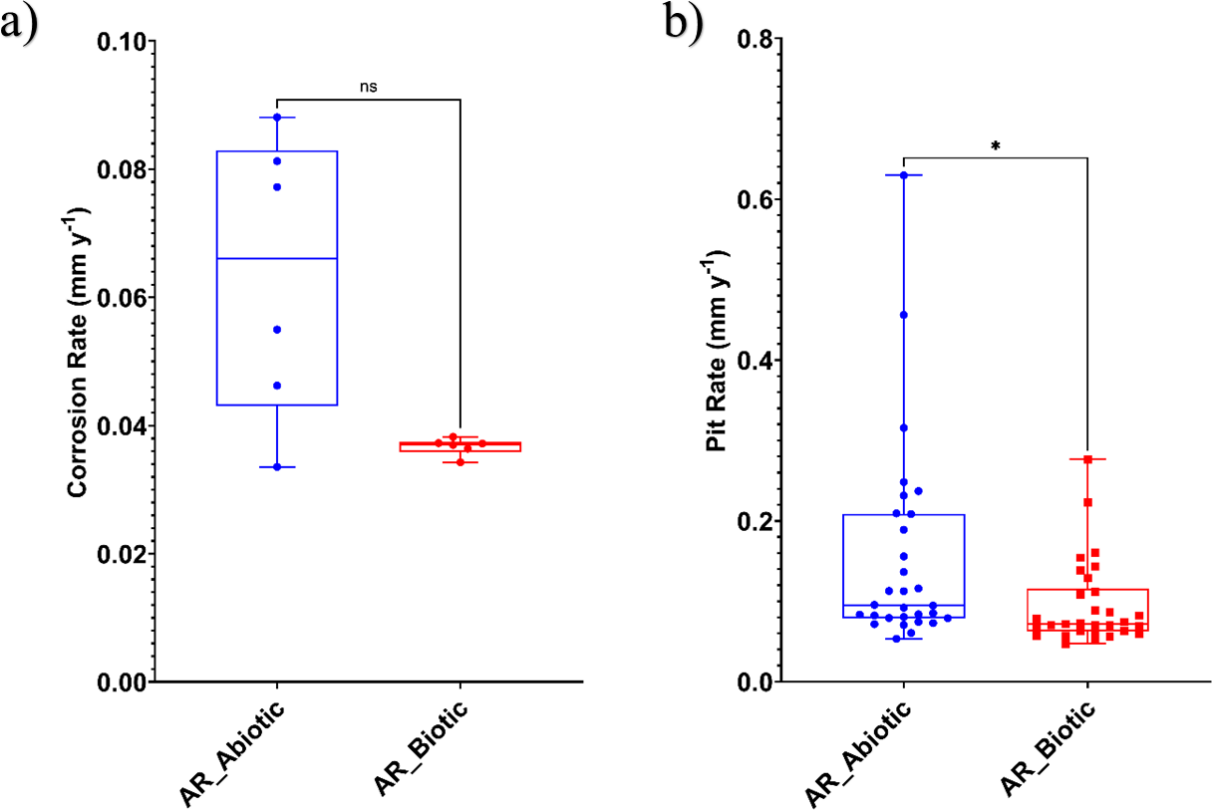
Abiotic and biotic corrosion performance after exposure to anaerobic-produced water media for 28 days: (a) corrosion rate measured via gravimetric analysis and surface profilometry, and (b) pit rate (*P* < 0.05) for the AR coupons.

Figure S4 shows the CS surfaces on day 0. Figure S5 summarizes the quantitative surface roughness profiles on both day 0 and day 28. Figure 1 shows the cleaned CS surfaces after 28 days, with biofilms and corrosion products removed to reveal the morphology of the surface degradation and facilitate corrosion assessment. Surface profilometry revealed that there were low levels of uniform or localized pitting corrosion present for both the abiotic and biotic conditions. The abiotic average pit depth was 12 µm, with an average pit area of 971 µm^2^ for any classified pits. The biotic average pit depth was 7 µm, with an average pit area of 1,501 µm^2^ for any classified pits. Again, for this study, a pit was classified as having a depth greater than 5 µm and an area greater than 650 µm^2^ (30)

**Fig 2:**
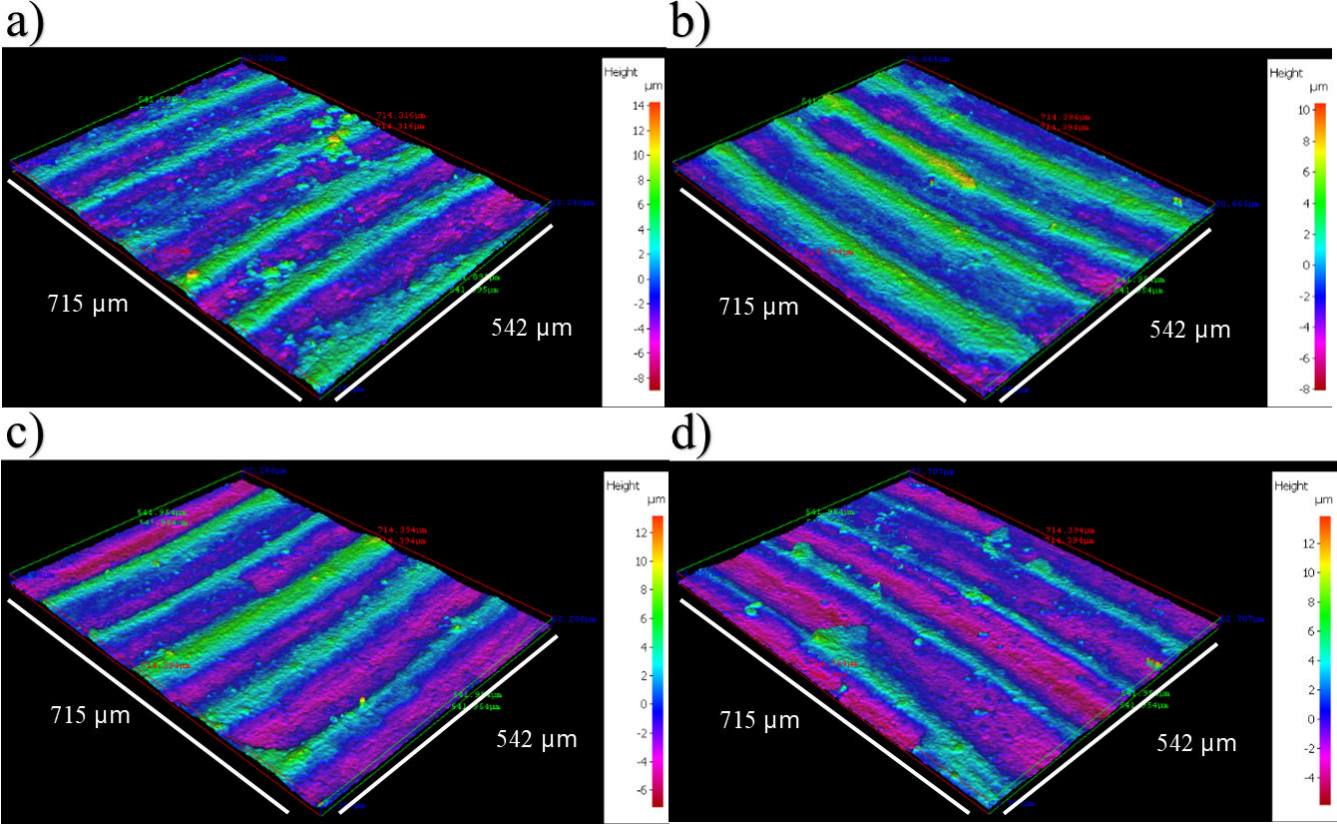
Three-dimensional optical surface profilometry of the cleaned UNS G10180 surfaces at day 28. AR coupons for (a and b) abiotic and (c and d) biotic conditions, after exposure to anaerobic produced water media for 28 days.

Figure 2a provides an evaluation of the CS coupons *CR*. For the abiotic condition, there was a higher *CR* when compared with the biotic condition, although there was no significance. According to the NACE SP0775-2023 assessment criteria, there was a moderate *CR* (between 0.025 and 0.12 mm y^–1^) in both the abiotic and biotic reactors (Fig. 2a). However, a moderate *PR* (0.13–0.20 mm y^–1^) was assessed for the abiotic condition, with a low *PR* (<0.13 mm y^–1^) for the biotic condition (Fig. 2b) (33). Further analysis of the surface profilometries in Fig. 1 allowed a quantitative determination of the *PR* Fig. 2b of the CS coupons. For this study, it was not possible to quantitatively determine *PD* values, due to low *PR* across the coupon surfaces upon retrieval after 28 days.

### Corrosion product analysis

**Fig 3:**
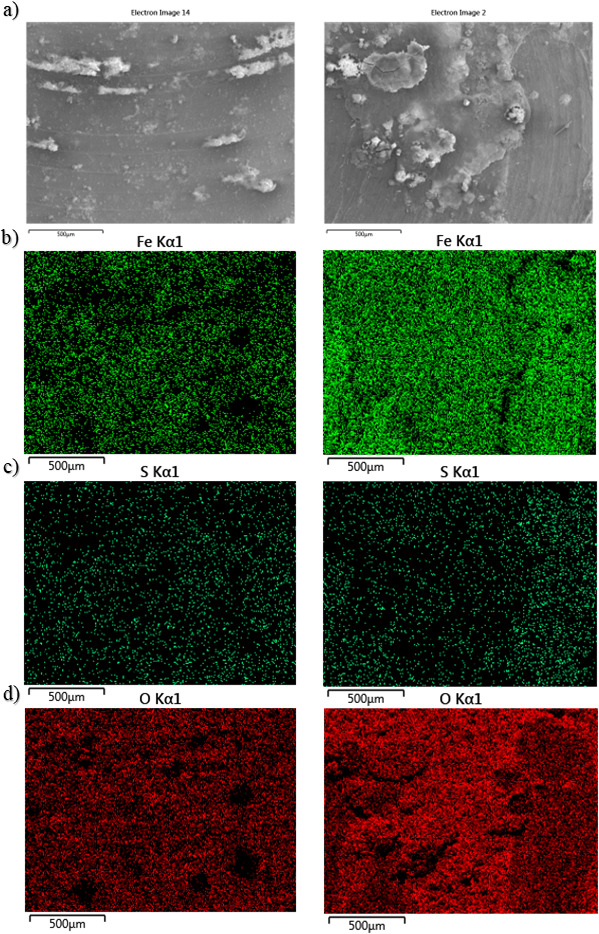
SEM-EDS elemental mapping of the UNS G10180 carbon steel, AR surfaces, after exposure to anaerobic produced water media, taken on day 28. (a) SEM image; (b) iron map; (c) sulfur map; and (d) oxygen map.

Figure 3 shows SEM-EDS elemental mapping of the UNS G10180 CS surfaces for both the abiotic and biotic conditions. Quantitative SEM-EDS data collected from elemental mapping are shown in Fig. S6. The images of corrosion products and biofilms attached to the metal samples demonstrate the heterogeneity of distribution over the surface. Coupons exposed to the biotic condition were observed to exhibit greater surface coverage. The SEM-EDS elemental maps are shown in Fig. 3b through d. The major elements detected in coupons exposed to all conditions were iron (Fe), sulfur (S), and oxygen (O). Corroded areas of all coupons were mainly covered by Fe and O, with heterogeneous distribution of S. A cross-sectional image of the corrosion products was not performed.

**Fig 4:**
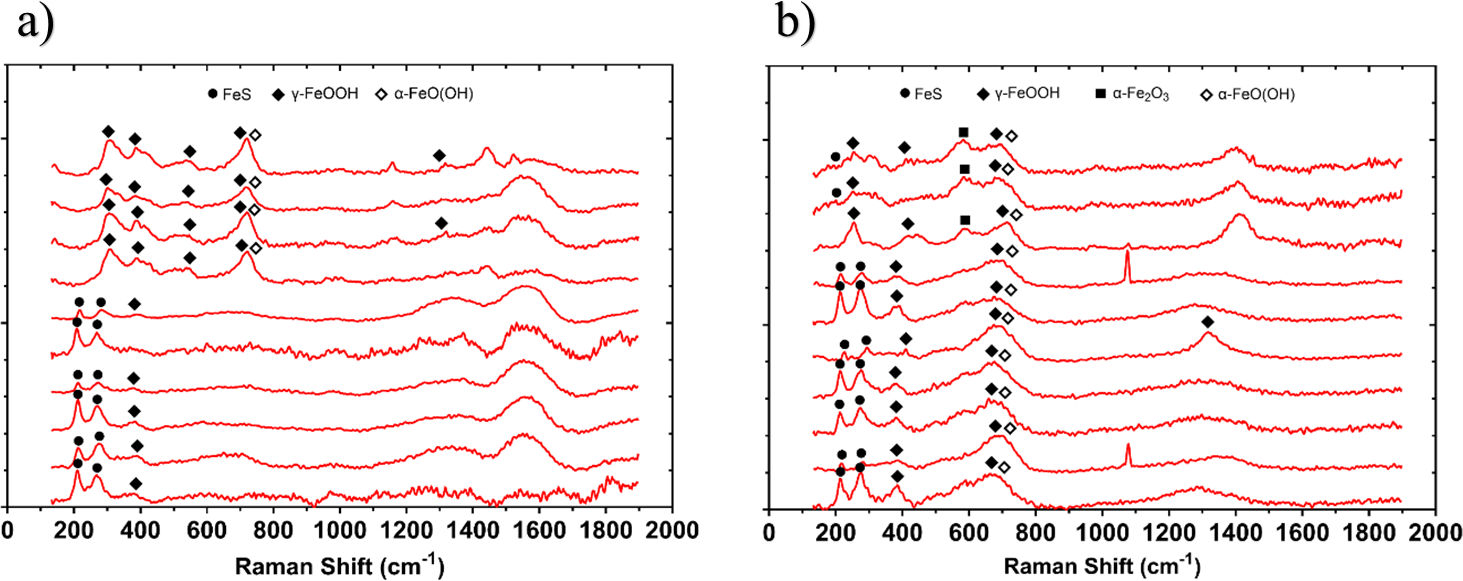
Raman spectra of the UNS G10180 carbon steel surfaces after exposure to anaerobic produced water media, taken on day 28. Shown are results for the AR (a) abiotic and (b) biotic conditions.

Additional analysis of the corrosion products using Raman spectroscopy is shown in Fig. 4. According to Raman bands of reference corrosion products in previous papers (34–36), the corrosion products are identified to be primarily mackinawite (bands 208, 282 cm^–1^) for both the abiotic and biotic conditions. There were also additional bands that may be attributed to sulfur, as well as reference iron oxide compounds such as magnetite, goethite [α − FeO(OH)], lepidocrocite, or hematite (α − Fe_2_O_3_). The composition of this black compact layer was identified at mid-strong bands 250, 380, and 1307 cm^–1^ associated with lepidocrocite. Additionally, bands at 298, 399, 481, 554, 675, and 1,002 cm^–1^ have previously been shown to be associated with goethite, whereas bands at 222, 244, 298, 501, 615, and 1318 cm^–1^ are associated with hematite. Magnetite has previously been shown to be associated with bands at 675 and 550 cm^–1^ (34–36). The coverage of the metal sample with a black precipitate was indicative of the successful growth of corrosion products film containing FeS compounds. The Raman spectrum of the sample is in good agreement with literature spectra attributed to mackinawite.

### Electrochemical measurements

**Fig 5:**
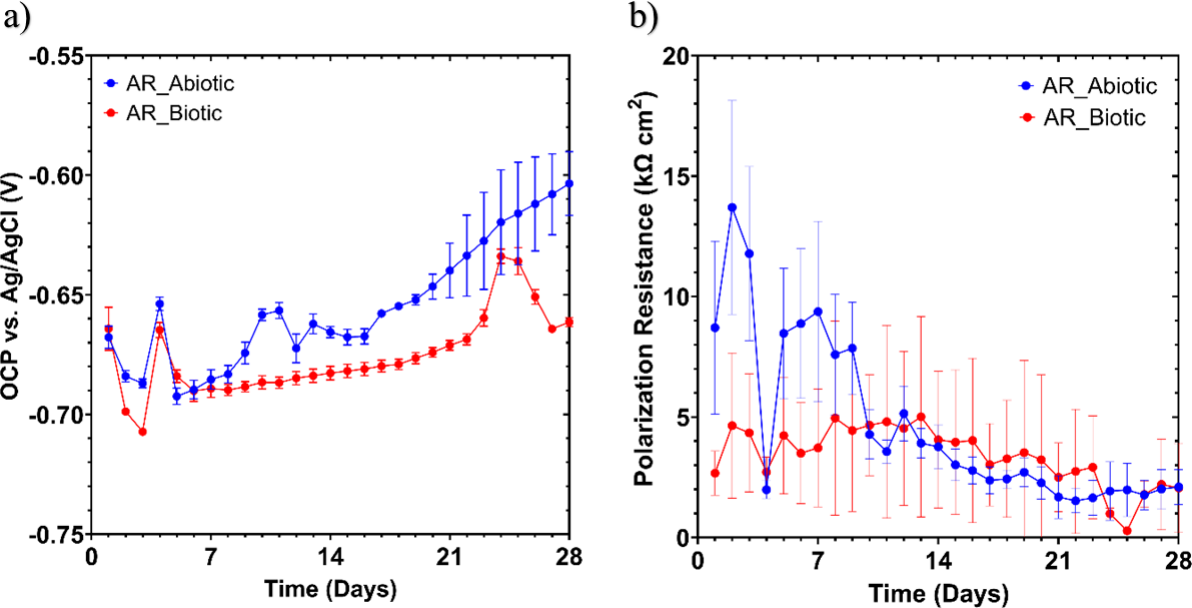
LPR data for UNS G10180 carbon steel: (a) open-circuit potentials and (b) polarization resistance in anaerobic produced water media (abiotic and biotic conditions) for AR and P coupons (data points represent mean ± standard deviation, *n* = 6). Reactor stirrer at 50 rpm.

Figure 5 shows the changes in *E*_corr_ and *R*_p_ between the abiotic and biotic anaerobic PW media for the UNS G10180 CS coupons. For the abiotic condition, Fig. 5a, there was a swift increase of +0.040 V on day 3 after the end of the batch phase. This was subsequently followed by a swift decrease of −0.050 V on day 4. This can be attributed to the flow of fresh PW. Otherwise, there was a gradual +0.090 V electronegative shift in the *E*_corr_ between days 4 and 28. This can be linked with the presence of a conditioning film (i.e., an adsorbed organic layer) and the formation of inorganic corrosion product layer. A pseudo-steady state *E*_corr_ had not been attained after 28 days. Similarly, for the biotic condition, there was a swift increase of +0.050 V on day 3 after the end of the batch phase. This was subsequently followed by a swift decrease of −0.030 V on day 4. Then, there was a gradual electronegative shift in the *E*_corr_, until day 24. After which, the *E*_corr_ swiftly decreased by −0.040 V. The potentials for both abiotic and biotic in the latter stages were generally similar and ranged between –0.610 V and –0.670 V vs. Ag/AgCl.

In Fig. 5b, the LPR-derived *R*_p_ after day 10 remained low at approx. 400 Ω cm^2^ for the sterile abiotic condition, indicative of uniform corrosion across a porous corrosion film. Similarly, for the biotic condition, the *R*_p_ remained low at approx. 400 Ω cm^2^. The pioneering bacterial attachment/colonization was difficult to detect for this study. However, biofilm formation and growth kinetics will lead inevitably to a more complex electrochemical response. Overall, there were no significant differences when comparing between the abiotic and biotic reactor environments.

**Fig 6:**
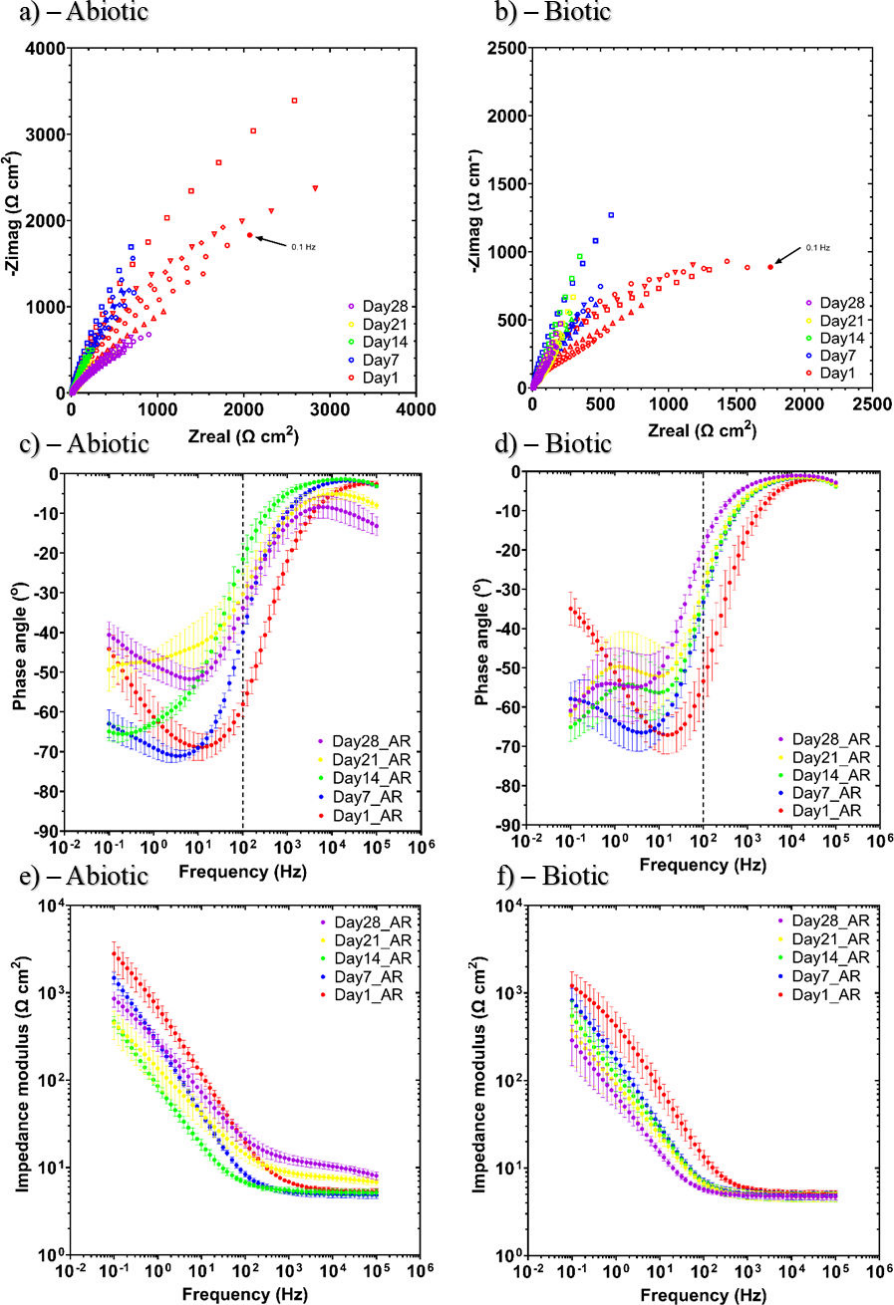
EIS data for UNS G10180 carbon steel in anaerobic produced water media at OCP: (a and b) Nyquist, (c and d) Bode phase angle (θ vs*. f*), and (e and f) Bode impedance modulus (|Z| vs*. f*) over 28 days (*n* = 6). Reactor stirrer at 50 rpm.

Figure 6 shows the EIS data for UNS G10180 CS in the anaerobic PW media presented in three forms: Nyquist, Bode phase angle, and Bode impedance modulus plots. The sterile abiotic condition on day 1 typifies an electrochemical response for the formation of a porous interface, with diffusion of soluble electroactive species across an organic conditioning film (37) and a nascent inorganic corrosion product layer. The diffusive behavior is associated with linear features having a roughly 45° slope (a Warburg impedance response) and phase angles close to 45° in low-frequency region (10^–2^ to 10^0^ Hz), see Fig. 6a and c. This electrochemical response did not really change over the 28 days. Similarly, the biotic condition had a consistently uniform EIS response over the 28 day test, with only a minor variation in the spectra and suggests that the absence of significant detectable electrochemical changes with time. Notably, there are no discernible Nyquist semicircles (Fig. 6b). Here, a wider low-frequency region (10^–1^ to 10^2^) is likely to be subject to a greater influence of adsorption processes, associated with the adhesion of the pioneering bacteria on a conditioning film (38, 39) and biofilm formation. The EIS spectra were fitted using an equivalent circuit model (ECM) shown in Fig. S7. Both the abiotic and biotic data generally had a good fit, with the quantitative fitting results shown in Table S8 . *R*_s_, *R*_film_, and *R*_ct_ are the solution resistance, the resistance of the biofilm or the corrosion product film, and the charge transfer resistance, respectively. The constant phase element (CPE) characterizes the “non-ideal” capacitance behavior of either the biofilm or the corrosion product film layer, and the charge transfer capacitance. In the Table S8, Q and n are admittance and exponent parameters from the CPE.

For the abiotic control, there is a capacitive behavior over the first week, with a diffusive behavior over the final 3 weeks in the film layer (reflecting ion adsorption). However, there was a diffusive behavior in the double layer, which reflects charge transfer, due to the formation of corrosion products (rust, porous oxide layer). The exponent parameter in the double layer reflects a non-ideal capacitance, which is indicative of resistive and inductive parasitics, because of a more prominent resistive component. *R*_film_ is relatively low over the 28 days, and there are no significant changes in *R*_ct_. Similarly, for the biotic condition, there was a capacitive behavior over the first week, with a diffusive behavior over the final 3 weeks in the film layer. However, there was a diffusive behavior over the initial 2 weeks, followed by a capacitive behavior over the final 2 weeks in the double layer. This capacitive behavior is attributed to the biofilm. There are no significant changes in the *R*_ct_ in the double layer over time. The exponent parameter for the film layer is greater than 0.8 only on day 28, which indicates a non-ideal capacitance response. This is true for the final 2 weeks in the double layer. The ECM and EIS both have general agreement with the LPR data.

Figure S9 shows the potentiodynamic polarization curves for UNS G10180 CS for the abiotic and biotic reactors in anaerobic PW media after 28 days. Table S10 shows the corrosion parameters obtained from the polarization curves. From the Tafel slopes, there is a similar cathodic behavior (reduction) when comparing the abiotic and biotic conditions, which is linked to the predominant HER under anaerobic conditions. Conversely, the anodic Tafel slopes (oxidation) are greater, demonstrating almost limiting current densities in the abiotic compared with the biotic media. Overall, the abiotic condition had a higher *j*_corr_ compared with the biotic condition. This is consistent with a more uniform corrosion morphology. Similarly, the sterile abiotic condition had a more electropositive *E*_corr_ when compared with the biotic condition. The polarization results corroborate the LPR and EIS data.

### Biofilm characterization

CLSM with differentiation of live and dead biofilm cells was performed and can be found in the supplementary material Fig. S11. The heterogeneous biofilm distribution over the surface of the CS coupons did not allow measurements of the maximum biofilm thickness. Therefore, the thickness of biofilms was not determined. From the images captured, there was a live/dead cell ratio of approximately 87% live to 13% dead.

**Fig 7:**
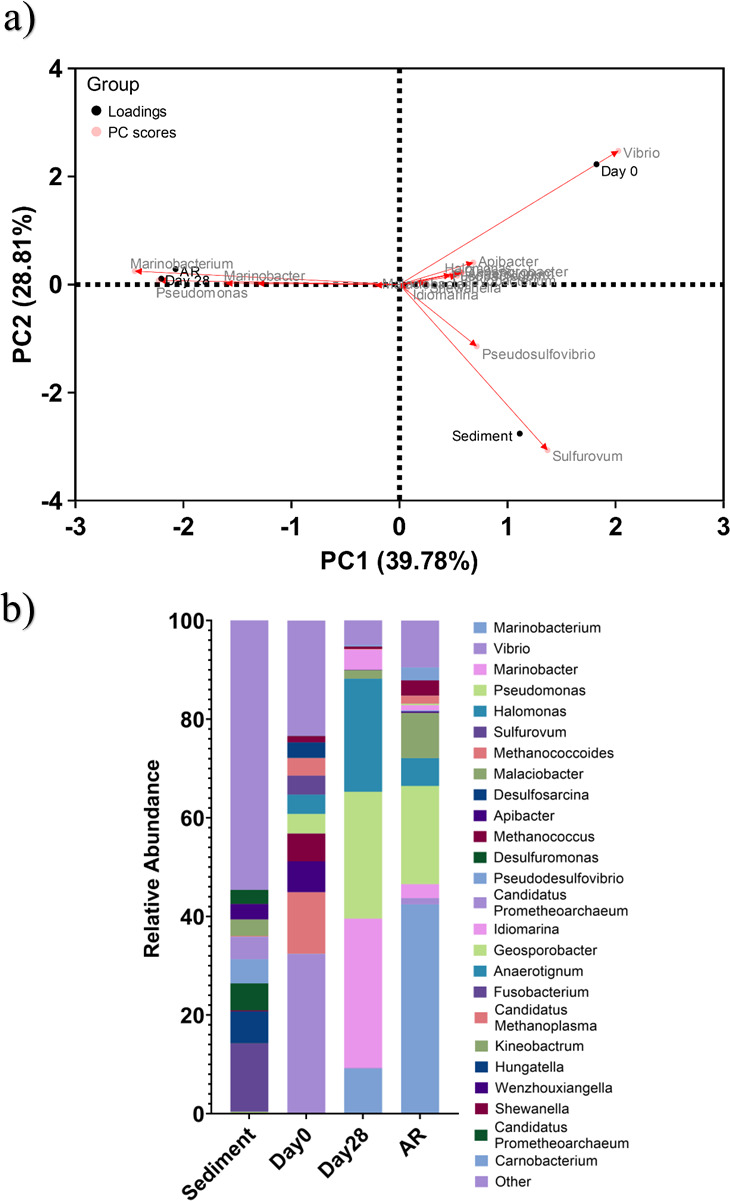
Principal component analysis biplot (a); microbial community. The results show the mean relative abundances of microbial communities classified at the genus level, for the top 25 genera, from 16S rRNA amplicon sequencing (b); for environmental marine sediment, day 0, and day 28 planktonic samples, and AR biofilms, after exposure to anaerobic produced water media for 28 days.

Active microorganism evaluation of the environmental marine sediment, the initial and final biotic PW media planktonic samples (day 0 and day 28), and the biotic AR biofilm were undertaken via 16S rRNA amplicon sequencing with two target regions, V3 and 4 for bacteria and archaea. A total of 2,293,909 high-quality sequences were obtained after bioinformatics processing of the raw reads. Among these samples, 97.7% were classified for the sediment sample, 99.99% for the day 0 planktonic sample, and 100% for the day 28 planktonic and AR biofilm samples. These sequences were taxonomically classified into microbial genera. The top 25 microbial genera are presented in Table S12 in the supplemental material. Fig. 7 summarizes the sequencing data, showing a PCA (a) and a stacked bar plot (b) illustrating the relative abundances for the top 25 genera. Molecular identification of the microorganisms showed that the initial sediment sample had a very diverse microbial composition. Most genera had low relative abundances less than 2%. The dominant genera included *Sulfurovum*, *Candidatus Prometheoarchaeum*, *Desulfosarcina*, *Desulfuromonas*, and *Thiohalobacter*. Interestingly, there were relatively high numbers of archaea in the sediment sample compared with other samples.

The sediment sample had negative Spearman correlation coefficients (Fig. S13) compared with the other samples, which was attributed to changes in conditions such as temperature and media composition from the natural marine environment. There was much less diversity in the day 0 sample, with *Sulfurovum*, *Candidatus Prometheoarchaeum,* and *Thiohalobacter* all exhibiting negligible relative abundances. However, genera from *Vibrio*, *Methanococcoides*, *Apibacter*, and *Methanococcus* made-up approximately 55% of the relative abundance. Again, the day 0 planktonic sample had negative Spearman correlation coefficients compared with the other samples. After 28 days, there was a significant shift in the microbial composition, with substantially lower abundances of methanogenic species. Conversely, there was a significant increase in Proteobacteria species. *Marinobacter*, *Pseudomonas*, and *Halomonas* were the dominant genera, making up approximately 80% of the relative abundance. The day 28 planktonic sample had a Spearman correlation coefficient of −0.43 compared with the sediment sample, −0.38 compared with the day 0 planktonic sample, and 0.89 compared with the AR biofilm sample. The relative abundances of *Vibrio* decreased to approximately 1%, with the relative abundances of *Methanococcoides*, *Apibacter*, and *Methanococcus* also decreasing to negligible values in the AR biofilm. Moreover, *Sulfurovum*, *Candidatus Prometheoarchaeum*, *Desulfosarcina*, *Desulfuromonas,* and *Thiohalobacter,* which were the dominant genera from the sediment sample, all had negligible relative abundances in the biofilm sample. The dominant genera included *Marinobacterium*, *Pseudomonas*, and *Malaciobacter,* making up approximately 70% of the relative abundance. There were no methanogenic archaea in the biofilm sample.

The microbial activity was determined by the ATP concentrations (dissolved, dATP) in the bulk fluid, see Fig. S14. The ATP assay did not measure any ATP from the biofilm sample. The biofilm may have been loosely adherent to the coupon surfaces or may have been rinsed off during sample preparation. For the biotic PW media (bulk fluid), there was a significant change (*P* < 0.05) in the dATP concentration when comparing day 0 and day 28, with dATP values on the order of 100 pg mL^–1^. As expected, there was a significantly greater ATP concentration for the biotic compared with the abiotic condition.

## DISCUSSION

Case studies from offshore oilfield systems have consistently demonstrated that environmental factors such as temperature, salinity, $O_{2}$ levels, and the composition of PW play a crucial role in the development of biofilms and the severity of MIC on CS surfaces (40, 41). These studies underscore the importance of implementing tailored corrosion management strategies that include regular monitoring, biocide application, material selection, and maintenance to mitigate the threat of MIC in offshore oil and gas operations. However, there are limitations and challenges when testing *in situ*. Offshore environments are often remote and difficult to access, making it challenging to collect samples and monitor corrosion processes over time. Environmental variability, including changes in temperature, pressure, salinity, and flow rates, can further complicate the identification of specific factors driving MIC. Continuous long-term monitoring is essential to fully understand the dynamics of biofilm development and MIC in offshore systems. However, maintaining long-term studies in such harsh environments is challenging. Consequently, this leads to a reliance on shorter-term studies that may miss important trends. This can make it difficult to identify and characterize the full range of microorganisms involved in MIC, as well as understand their interactions. These limitations underscore the need for more robust and innovative approaches to studying MIC using more realistic laboratory simulations of offshore environments.

**Fig 8:**
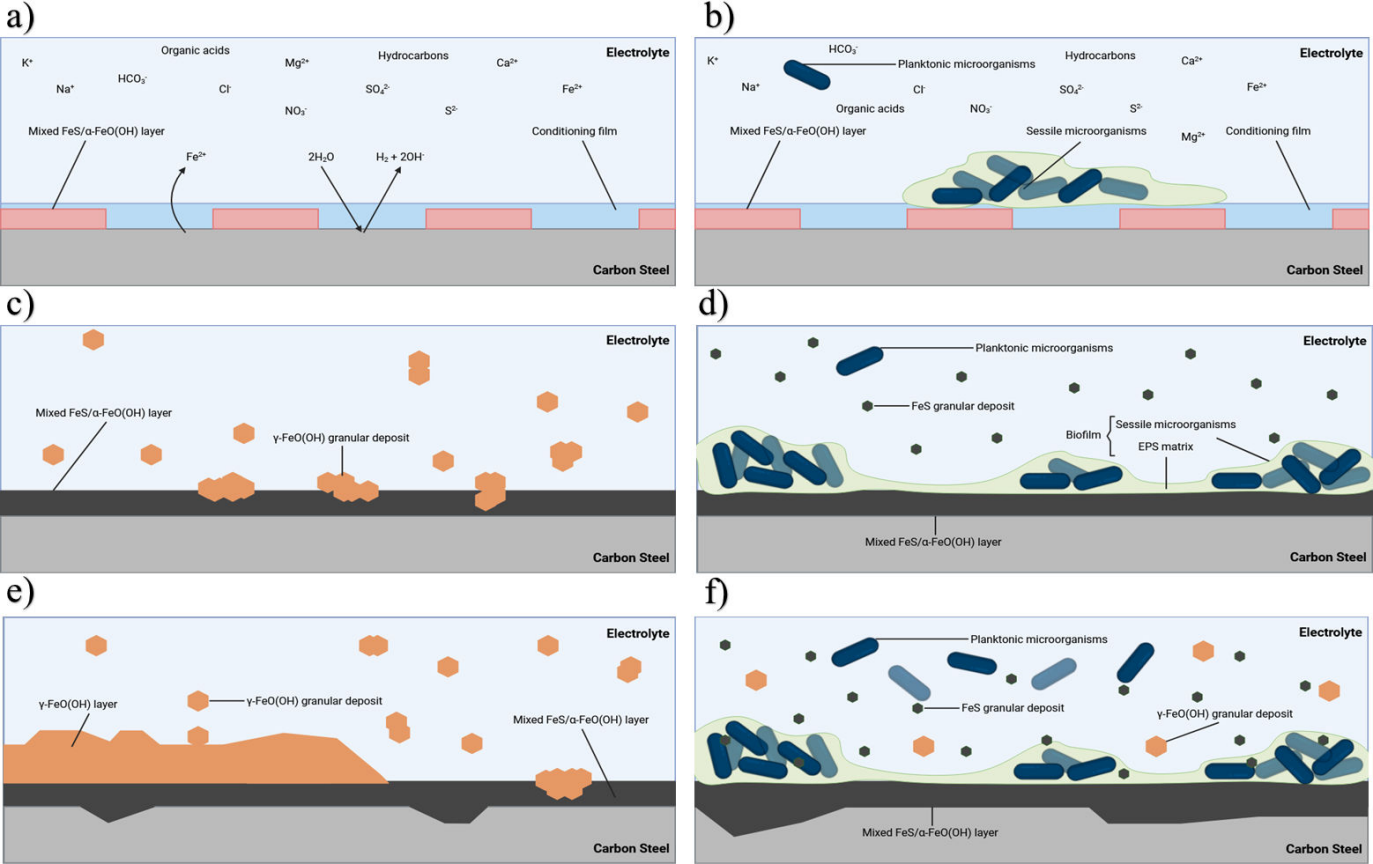
Illustration of the initial stages for UNS G10180 carbon steel in anaerobic abiotic and biotic PW media. Corrosion mechanisms, (a and b) the formation of nascent inorganic corrosion film and the organic conditioning film with pioneering bacterial attachment during the initial batch phase; (c and d) maturing corrosion film under the abiotic condition with reduced biofilm growth and colonization under the biotic condition due to the limited availability of organic carbon; (e and f) moderately low uniform and pitting corrosion under patchy corrosion deposits and thin biofilm with increasing granular deposits. BioRender.com (2024).

Figure 8 provides an illustration of the proposed corrosion mechanisms for both the abiotic and biotic conditions during the initial stages, as they evolved over time during this present study.

### Abiotic reactor

After the initial batch phase, there was a general electronegative shift associated with anodic polarization for the abiotic condition. This can be attributed to changes in the steel surface as it interacts with PW. This environment typically contains hydrocarbons, organic acids, salts, and inorganic compounds. These substances can adsorb onto the steel surface, forming a conditioning film of mixed organic and inorganic layers, as described in corrosion studies involving PW and seawater (42, 43). The anodic polarization reflects the active dissolution of iron, likely influenced by this initial conditioning layer. This behavior aligns with findings that organic and inorganic films alter electrochemical properties, affecting ion adsorption and leading to transient capacitive behavior (44). A pseudo-steady state *E*_corr_ had not been attained after 28 days. This suggests ongoing dynamic changes at the steel-water interface, driven by continued interaction with the conditioning film and subsequent corrosion product formation (43, 45). PW often contains various organic compounds, including hydrocarbons and organic acids (46). Additionally, PW typically contains inorganic compounds such as salts, minerals, and metal ions. Both organic and inorganic substances can precipitate on metal surfaces, contributing to the formation of a conditioning film. This film can affect the electrochemical properties of the steel surface. Thus, the conditioning film can play a dual role. It can either passivate the surface or facilitate corrosion. The observed capacitive behavior in the first week reflects ion adsorption processes, which reflects ion adsorption and the development of a conditioning film (mixed organic and inorganic interfacial layer). However, the subsequent diffusive behavior indicates charge transfer linked to corrosion product growth. Moreover, there was a diffusive behavior in the metallic interface double layer, which reflects charge transfer, due to the formation of corrosion products. The primary corrosion product identified was mackinawite. Under abiotic conditions, mineral formation is driven by purely chemical reactions between the artificial seawater and the CS coupons. Mackinawite typically forms under highly reducing conditions, and its presence suggests a chemical reaction between dissolved sulfide and iron ions (35, 36). Mackinawite is highly reactive and accelerates corrosion by creating localized galvanic cells. There were also additional bands, which may be attributed to sulfur, as well as reference iron oxide compounds such as magnetite, goethite, lepidocrocite, and hematite. Moreover, the heterogeneous distribution of sulfur and the presence of iron oxides (magnetite, goethite, and hematite) suggest the coexistence of anoxic and oxidizing microenvironments on the steel surface, facilitating diverse corrosion processes. These findings are consistent with the literature on biotic and abiotic contributions to steel corrosion in similar environments (45, 47). Additional analysis identified that corroded areas were mainly covered by Fe and O, with heterogeneous distribution of S. Generally, there was a moderately low level of uniform corrosion of the steel surface for the abiotic media.

### Biotic reactor

During the initial batch phase, where additional organics were available via supplementation, an electronegative shift in the *E*_corr_ was observed. This correlates with increased organic availability from the yeast extract supplementation, stimulating biofilm formation on the CS. It is important to note that yeast extract was only added in the pre-culture used to inoculate the biotic reactor. The bulk fluid used over the 28 days had no such supplementation and was only PW. A previous study found that omitting yeast extract from their medium led to a significant reduction in hydrogen sulfide production. However, this reduction did not correspond to a decrease in corrosion rates; in fact, an increase in corrosion was observed. This suggests that hydrogen sulfide production and sulfate reduction might not be the most critical metrics for MIC, indicating that other factors influenced by yeast extract omission could play a significant role in corrosion processes (48). Biofilms formed during this phase can concentrate sulfides, organic acids, and metabolites, altering the local chemical environment and impacting the *E*_corr_. This aligns with studies highlighting the role of organics and biofilm formation in influencing surface chemistry (42, 47). The visible changes in the medium (dark green/black coloration, black precipitates, and turbidity) suggest active microbial metabolism, possibly involving SRB. These microorganisms reduce sulfate to sulfide, which reacts with iron to form corrosion products like mackinawite, contributing to the observed coloration and precipitate formation (43). However, once the flow of fresh media was initiated, there was a similar electronegative shift, which was observed for the abiotic condition. The potentials for both abiotic and biotic conditions in the latter stages were generally similar. The similar potentials suggest that the overall system is dominated by the uniform distribution of corrosion products, particularly mackinawite under reducing conditions. This observation is consistent with reports of abiotic and biotic corrosion showing overlapping electrochemical trends under specific environmental conditions (43, 45). Additionally, the *R*_p_ was generally low for both conditions. The low *R*_p_ in both conditions indicates a high rate of charge transfer reactions, characteristic of active corrosion. For the biotic condition, there is initially a capacitive behavior observed over the first week, with a diffusive behavior over the final 3 weeks in the film layer. The capacitive behavior in the biotic condition during the first week reflects ion adsorption and biofilm formation, whereas diffusive behavior in later stages corresponds to corrosion product development. This was similar to the abiotic condition. In contrast to the abiotic condition, the double layer for the biotic condition showed inverse trends, with the capacitive behavior in later weeks attributed to biofilm formation, which may cause diffusion limitations (44, 47). Likewise, the formation of corrosion products, which were primarily identified as mackinawite, will also impact the possible electrochemical reactions that are taking place at the interface of the metal/electrolyte. Under biotic conditions, the presence of biofilm fundamentally alters the corrosion process due to the microbial activity of heterotrophic microorganisms. Mackinawite, the primary corrosion product, forms through chemical interactions under reducing conditions and is accelerated by SRB activity (49–51). This biofilm-driven process concentrates sulfides and creates localized microenvironments, potentially enhancing pitting corrosion. However, low levels of localized pitting observed in the study indicate the early stages of biofilm activity and corrosion progression (43, 47).

Surface profilometry analysis provided further insights and revealed that there were low levels of uniform or localized pitting corrosion present for both the abiotic and biotic conditions. However, after 28 days, the biotic condition did exhibit pits with a greater average area. This is characteristic of localized pitting caused by biofilms (52, 53). For this study, it was not possible to quantitatively determine *PD* values. Despite the low overall pitting levels, biofilms showed potential for localized corrosion progression over extended periods. The mixed-species biofilm’s synergistic interactions likely enhance microenvironment heterogeneity, accelerating localized corrosion in the long term.

Analysis of the community dynamics revealed a marked change in the predominant relative abundances of microorganisms. The dominant genera from the sediment sample were generally anaerobic, halophilic, and obligately chemolithoautotrophic, obtaining energy by oxidizing inorganic compounds. Interestingly, the relative total archaea from the day 0 planktonic sample still accounted for approximately 20% of the relative abundance. *Methanococcoides* and *Methanococcus* are both known methanogenic archaea that play an important role in the production of methane in anaerobic environments (54, 55). The primary metabolic pathway in *Methanococcus* involves the reduction of CO_2_ with H_2_ to form methane (54). However, *Methanococcoides* utilize methylotrophic methanogenesis, where methylated compounds serve as the primary substrates for methane production (55). *Vibrio* was the dominant genera in these samples at approximately 30% relative abundance and is typically found in marine and estuarine environments. *Vibrio* sp. are known for their ability to form biofilms on various surfaces, including metals (56). After 28 days, there was a significant increase in *Marinobacter*, *Pseudomonas*, and *Halomonas*, making up approximately 80% of the relative abundance. *Marinobacter* species are known for forming biofilms on metal surfaces in marine environments. These biofilms can influence the corrosion process by altering the local chemical environment (57). Moreover, *Marinobacter* species can degrade hydrocarbons, making them important for the bioremediation of oil spills in marine environments. They are well-adapted to high-salinity environments and play a role in iron cycling in marine environments, impacting the availability of iron (58, 59). *Pseudomonas* species are prolific biofilm formers, and these biofilms can enhance corrosion by creating microenvironments that promote differential aeration and localized corrosion (60). *Halomonas* species thrive in high-salinity environments and can reduce ${SO}_{4}^{2-}$ to H_2_S. Additionally, *Halomonas* species participate in nitrogen cycling, including denitrification, which is important for maintaining the balance of nitrogenous compounds in marine ecosystems (61). The AR biofilm sample also had relatively high abundances of Proteobacteria species, namely *Marinobacterium,* which made-up approximately 40%. Generally, the dominant species were halophilic or halotolerant and are known to be heterotrophic. They are well-adapted to marine conditions, playing roles in organic matter degradation, nutrient cycling, and interactions with marine organisms. Although the specific roles of *Marinobacterium* and *Malaciobacter* are less characterized compared with other bacteria like *Pseudomonas* or *Halomonas,* they occupy a similar phenotypic niche (62). Interestingly, there was an increase in the relative abundance of Shewanella within the AR biofilm. *Shewanella* species are known for their unique role in MIC, primarily due to their ability to reduce metal ions and interact directly with metal surfaces. In the presence of other bacteria, *Shewanella* can enhance corrosion through indirect mechanisms, such as H_2_S production and the formation of FeS. Moreover, *Shewanella* has demonstrated the ability for EET (21), which is an important process in MIC. It would be interesting to conduct longer-term studies with the same experimental setup to observe the threat of *Shewanella*.

For the conditions used in this study, more closely mimicking the environmental conditions of an offshore oilfield system, the abiotic surfaces had the presence of a black corrosion product across the entire coupon surfaces with some reddish-brown granular deposits. These products arise from localized chemical reactions driven by environmental factors, including temperature and the composition of artificial seawater (43, 47). As stated earlier, the primary corrosion product identified was mackinawite; with reference iron oxide compounds also detected. Mackinawite can form under anoxic or reducing conditions in environments containing H_2_S. H_2_S can be present in natural waters due to the reduction of ${SO}_{4}^{2-}$ by SRB. Moreover, mackinawite formation is favored in anaerobic environments. Conversely, iron oxides are typically formed in the presence of O_2_ and water through oxidation reactions. Several types of iron oxides can form, depending on environmental conditions. Initial electrochemical reactions produce iron hydroxides, which are subsequently oxidized further to iron oxides. Nonetheless, there appeared to be a passivation of the metal surface as *CR*s were moderately low. The moderately low corrosion rates and the apparent passivation reflect a balance between the protective and corrosive effects of the conditioning film and corrosion products. This is corroborated by the findings on under-deposit corrosion, where deposits and their associated films significantly influence the corrosion dynamics (42, 44). The described conditions mimic offshore oilfield systems, where the presence of mixed organic and inorganic constituents, coupled with anoxic environments, fosters similar corrosion mechanisms. The predominance of mackinawite and iron oxides aligns with documented evidence from offshore facilities experiencing MIC and abiotic corrosion (43, 47).

**Fig 9:**
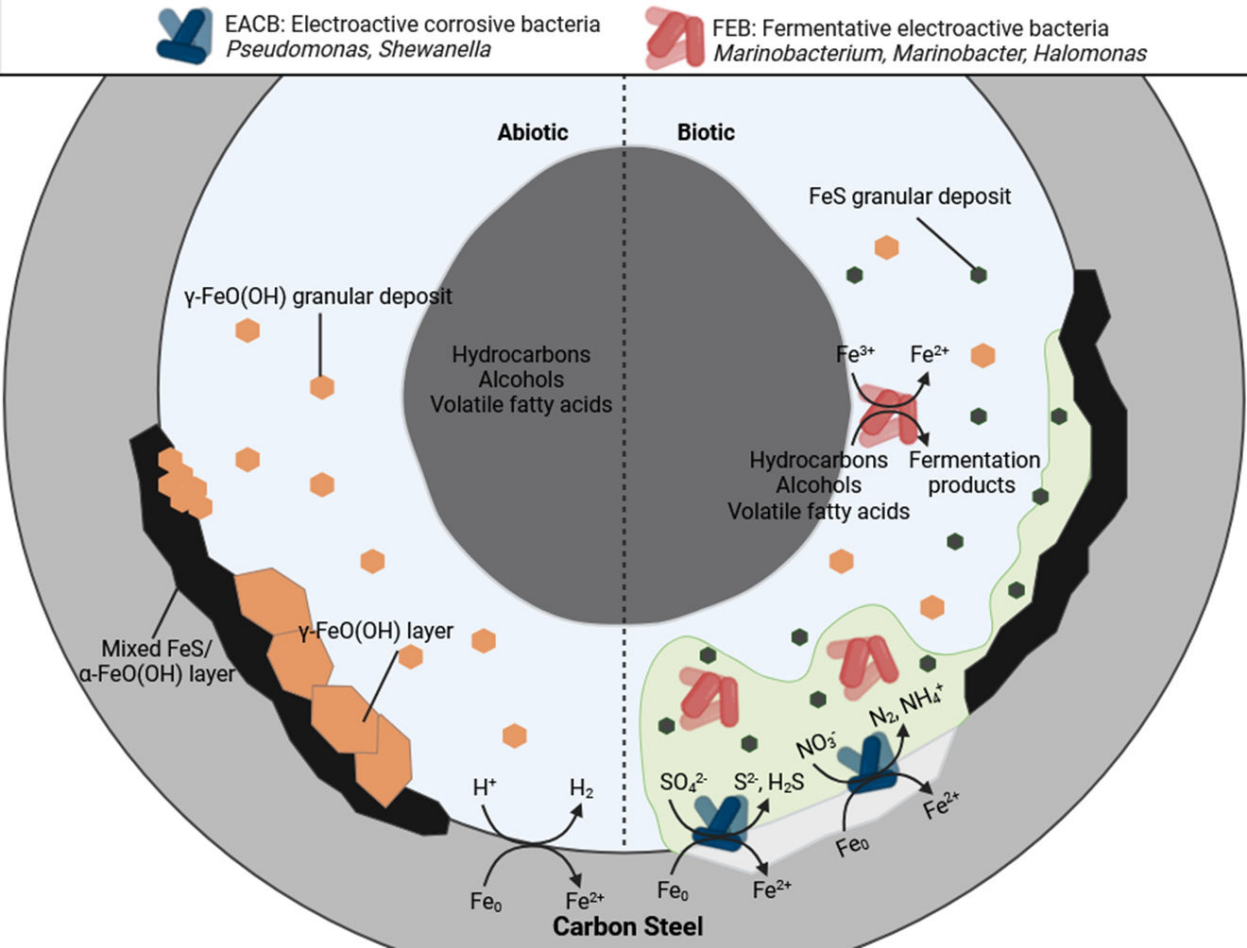
Overview of key reactions for UNS G10180 carbon steel in anaerobic abiotic and biotic PW media. It is important to note that the reactions illustrated may be complementary or antagonistic processes, particularly within the heterogeneous biofilm.

For this study, the electrochemical response under biotic conditions was not too dissimilar to the abiotic condition. However, surface observations and analysis of the biofilm suggest a different scenario. The biotic surfaces were only partially covered by a black corrosion product, with the presence of a heterogeneous dark green/black biofilm. The biofilm provides partial passivation through diffusion limitation while also promoting differential aeration, enhancing localized corrosion risks over time. The complex interplay of biofilm composition and metabolite production can greatly influence corrosion dynamics, as the biofilm acts as a diffusion barrier, limiting oxygen penetration and altering redox conditions at the metal interface (43, 47). As stated earlier, the mixed-species biofilm contained *Marinobacterium*, *Malaciobacter*, *Pseudomonas*, and *Halomonas,* all of which contribute to biofilm formation in high-salinity marine environments*.* They are important players in nutrient cycling within marine ecosystems and are crucial in creating microenvironments. In mixed microbial communities, these bacteria can work synergistically by altering environmental conditions (58–61). Through the production of organics and other metabolites, which can influence the redox potential or pH*,* they can create microenvironments, which may support ${SO}_{4}^{2-}$ and Fe^0^ reduction over long-term studies. It is hypothesized that the mixed-species biofilm is acting as a diffusion barrier and providing some passivation of the metal surface. Figure 9 proposes possible abiotic and biotic reaction mechanisms, associated primarily with the formation of mackinawite.

Previous laboratory studies have generally found that environmental conditions that mimic offshore oilfield systems, such as salinity, temperature, nutrient levels, and fluctuating O_2_ concentrations, have a profound impact on the formation, structure, and threat of corrosion from mixed-species biofilm communities on CS (44, 63, 64). These studies emphasize the importance of replicating real-world offshore conditions in laboratory experiments to accurately assess and mitigate the threat of MIC in oil and gas systems. However, like *in situ* studies, there are limitations and challenges when it comes to designing laboratory studies. One of the primary challenges in these studies is accurately replicating the complex and dynamic environmental conditions found in offshore systems, such as fluctuating temperatures, pressures, and chemical compositions. Laboratory settings often simplify these conditions, which can limit the relevance of the findings to real-world applications. Many studies are constrained by time, making it difficult to observe long-term biofilm development and the full extent of MIC. Corrosion is a slow process, and short-term studies may miss critical stages of biofilm maturation and corrosion progression. Maintaining a stable and representative microbial community in laboratory settings is also challenging. In industrial environments, microbial communities are constantly evolving, and this dynamic nature is difficult to capture in a controlled environment. Laboratory studies often struggle to replicate the scale and flow conditions of actual pipelines. The differences in flow dynamics can significantly impact biofilm formation and corrosion patterns, meaning that the lab results may not fully translate to field conditions. Thus, designing experiments that closely mimic real-world conditions as much as possible is critical. Furthermore, these findings highlight the importance of microbial community dynamics and biofilm interactions in MIC and emphasize the need for prolonged studies to understand critical corrosion progression phases. Long-term studies would likely reveal key insights into biofilm maturation, leading to increased corrosion severity and enhanced localized pitting driven by electroactive and biofilm-forming microorganisms. The contributions of key species through key mechanisms, such as EET could also be revealed.

### Conclusions

In conclusion, by utilizing a marine sediment microbial consortium and replicating the environmental conditions of an offshore oilfield system within the laboratory, we gained valuable insights into biofilm development, community dynamics, and the potential for inducing MIC within a novel dual bioreactor protocol.

- The electrochemical response under both abiotic and biotic conditions was found to be similar. An initial conditioning film appeared to form, influencing the electrochemical properties of the steel surface. However, the PW in this study did not support rapid biofilm growth, likely due to the absence of carbon-rich sources such as amino acids, peptides, and sugars.
- Mackinawite was identified as the primary corrosion product. Additionally, there were bands that may correspond to sulfur and reference iron oxide compounds, including magnetite, goethite, lepidocrocite, and hematite, observed under both conditions.
- Both conditions exhibited a moderately low uniform *CR* and limited localized pitting. Consequently, it was challenging to quantitatively assess *PD* values, due to low *PR* across the coupon surfaces. However, the biotic condition did display pits with a larger average area, indicative of MIC.
- Sequencing-based biofilm characterization revealed that *Marinobacterium*, *Malaciobacter*, *Pseudomonas*, and *Halomonas* played critical roles in biofilm formation under conditions simulating an offshore oilfield system. These microorganisms are essential in nutrient cycling within marine ecosystems and contribute to the creation of microenvironments.

This study begins to bridge the gap between experimental findings and real-world scenarios involving mixed-species biofilms and MIC. The innovative dual bioreactor protocol, which leverages MLOE, enables a comprehensive understanding of initial biofilm formation and the metabolic changes occurring within the biofilm over time. Identifying and characterizing specific microorganisms under simulated environmental conditions is crucial for understanding the threat posed by MIC. If not detected and mitigated early, the microbial mechanisms can lead to significant costs. Ultimately, the aim is to enhance the effectiveness of biofilm management strategies in the industry, thereby improving sustainability.

## Data Availability

The sequencing data generated in this study have been deposited in the NCBI Sequence Read Archive (SRA) under the accession numbers SAMN46521378, SAMN46521379, SAMN46521380, and SAMN46521381. Additional data supporting the findings of this study are available from the corresponding author upon reasonable request.

## AFTER EPUB

*[This article was published on 4 March 2025 with Niall Hanrahan missing from the byline, as well as errors in Materials and Methods and figure order. The errors were corrected in the current version, posted on 8 May 2025.]*

## SUPPLEMENTAL MATERIAL

Supplemental material aem.02221-24-s0001.pdf Supplemental tables and figures.

ASM does not own the copyrights to Supplemental Material that may be linked to, or accessed through, an article. The authors have granted ASM a non-exclusive, world-wide license to publish the Supplemental Material files. Please contact the corresponding author directly for reuse.
