# Supplementary material for: Toward simulating offshore oilfield conditions: insights into microbiologically influenced corrosion from a dual anaerobic biofilm reactor
Source: Appl Environ Microbiol. 2025 Mar 4;91(6):e02221-24. doi: 10.1128/aem.02221-24 (PMC12175496; doi:10.1128/aem.02221-24)
Supplement: Supplemental material — Supplemental tables and figures. [file aem.02221-24-s0001.pdf]

Supplementary Material

Table 1a. Produced water test solution/media composition. The produced water was filter sterilized using a 0.2 µm Vivaflow® TFF Cassette, PES (Sartorius).

Major Ion	Concentration (mg L^{-1})	Anions	Concentration (mg L^{-1})
Barium, Ba	141	Ammonium in water	54
Boron, B	73.4	Bicarbonate, HCO_3^-	642
Calcium, Ca	982	Bromide, Br^-	226
Chloride, Cl^-	31300	Chloride, Cl^-	31600
Iron, Fe	4.2	Fluoride	4
Magnesium, Mg	518	H2S dissolved in water	0.67
Potassium, K	259	Iodine, I^-	113
Sodium, Na	19100	Nitrate, NO_3^-	2
Strontium, Sr	96.5	pH at 20°C	6.7
Sulphate, SO_4^{2-}	750	Resistivity	0.136
Sulphur, S	523	Resistivity at temperature	22.4
Suspended solids	16	Specific gravity at 15°C	1.03868
Total dissolved salt	53300	Sulphate, SO_4^{2-}	550
Ionbalance (cation/anion)	1.9	Total alkalinity	19.5

Table 1b. Produced water test solution/media composition.

Organic Acids	Concentration (mg L^{-1})
Acetic acid	820
butanoic acid	15
Formic acid	12
Hexonic acid	<2
Pentoic acid	4
Propanoic acid	74

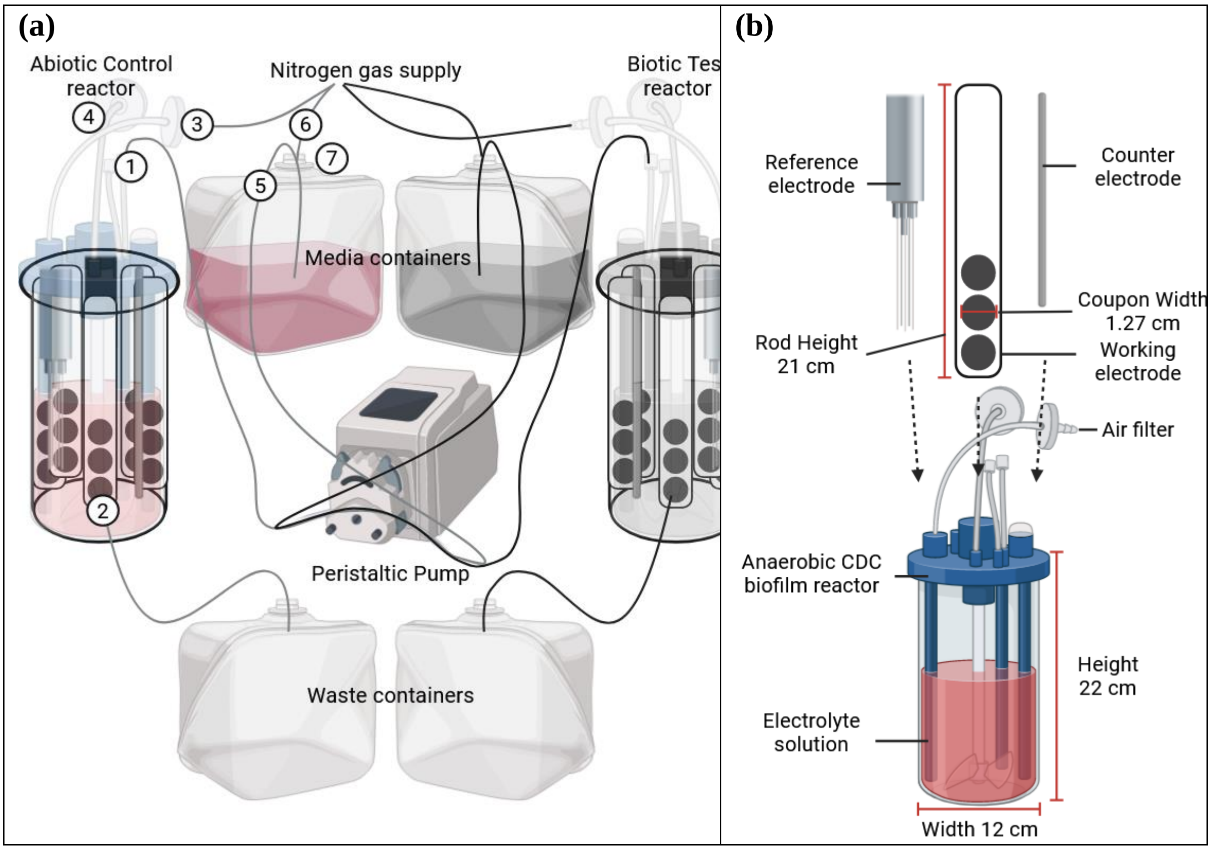

Figure 2. (a) The dual anaerobic biofilm reactor system (abiotic and biotic reactors) comprising 10 L media containers, peristaltic pump, magnetic stirrer/hot plate, sulphide microsensor, and the three electrode cell setup. Each reactor has five rods, with three coupons in each rod (15 coupons in total). Each reactor has four inlets. ① The first inlet is connected to the peristaltic pump and then the media container. ② The outlet is connected to the waste containers. ③ Connection to the nitrogen gas source. ④ Air filter (Millex, 0.2 µm) that acts as the exit for excess gas in the reactors. ⑤ The 10 L media container is connected via the peristaltic pump and feeds the first inlet in the reactor. ⑥ Connection to the nitrogen gas source. ⑦ Air filter (Millex, 0.2 µm) that acts as the exit for excess gas in the media containers. **(b)** detailed three-electrode cell setup in an anaerobic CDC biofilm reactor: There are three separate carbon steel coupon working electrodes that can be measured per rod. Each reactor had two rods that were modified for electrochemical analysis, *n* = 6 for as-received (AR) UNSG10180 carbon steel coupons. Created by BioRender.com.

Abiotic Coupons

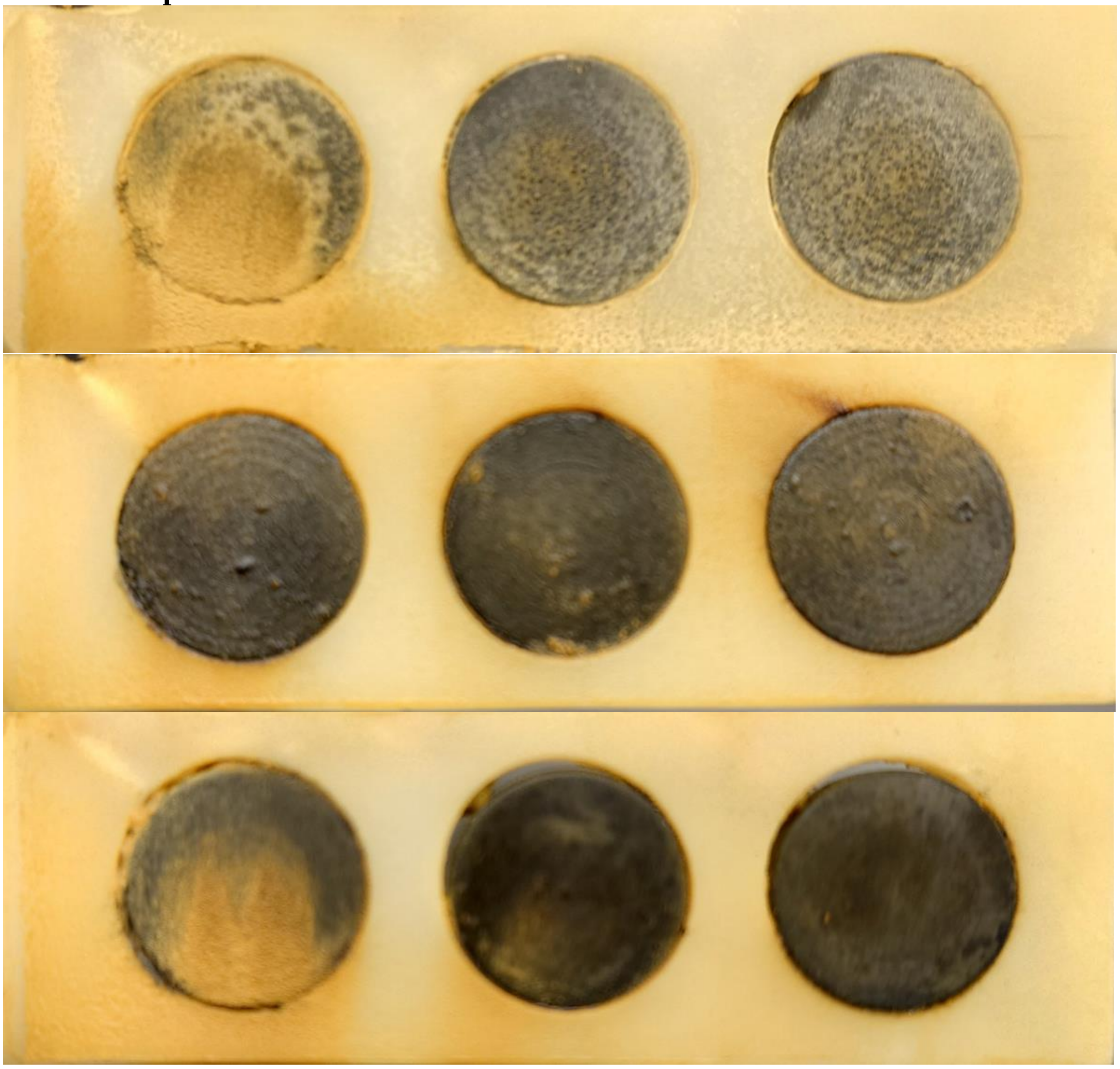

Figure 3a. Photographs taken of the coupon rods taken from the abiotic condition on Day 28, on dismantling the reactor, after exposure to anaerobic produced water media for 28 days.

Biotic Coupons

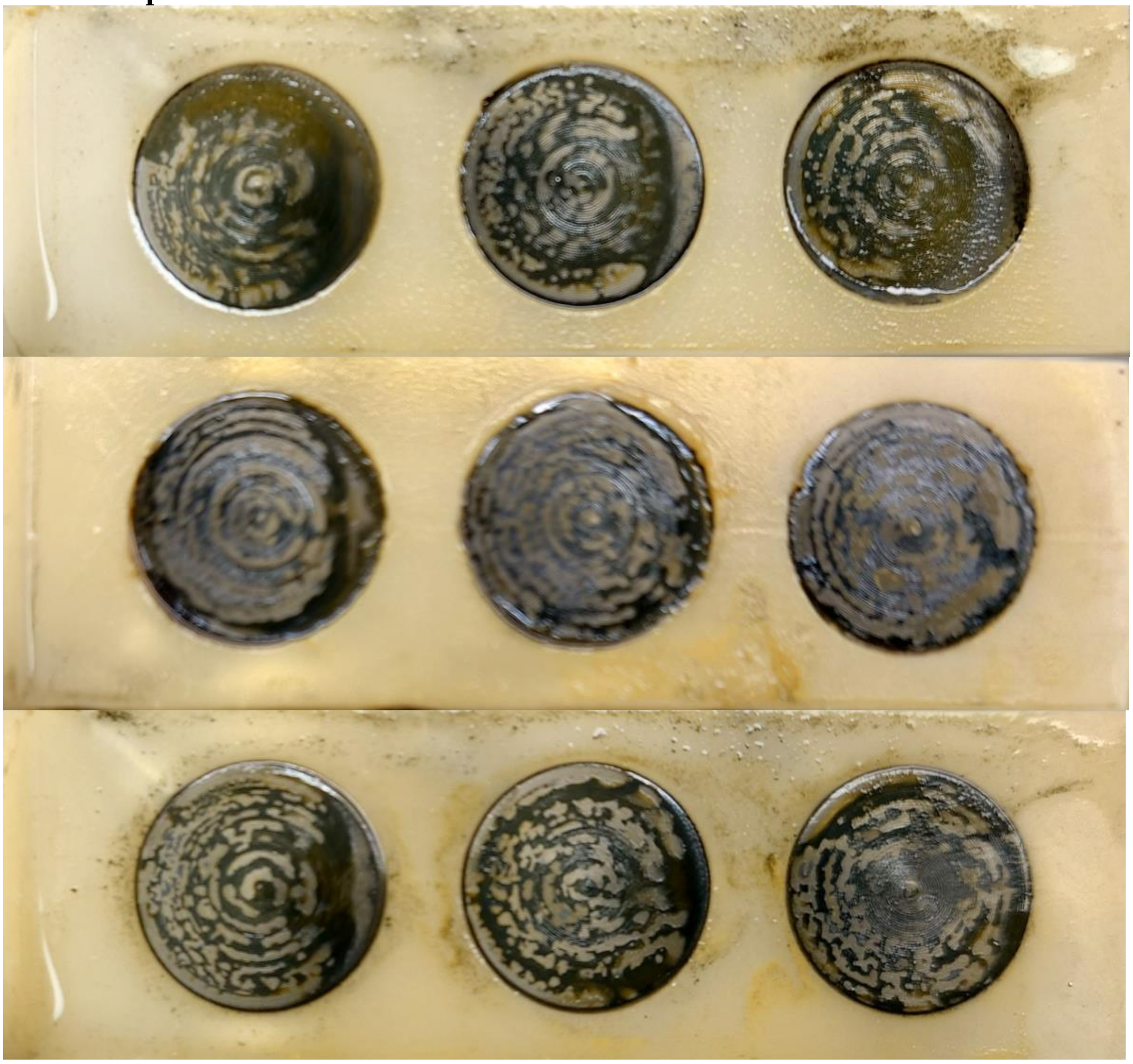

Figure 3b. Photographs taken of the coupon rods taken from the biotic condition on Day 28, on dismantling the reactor, after exposure to anaerobic produced water media for 28 days.

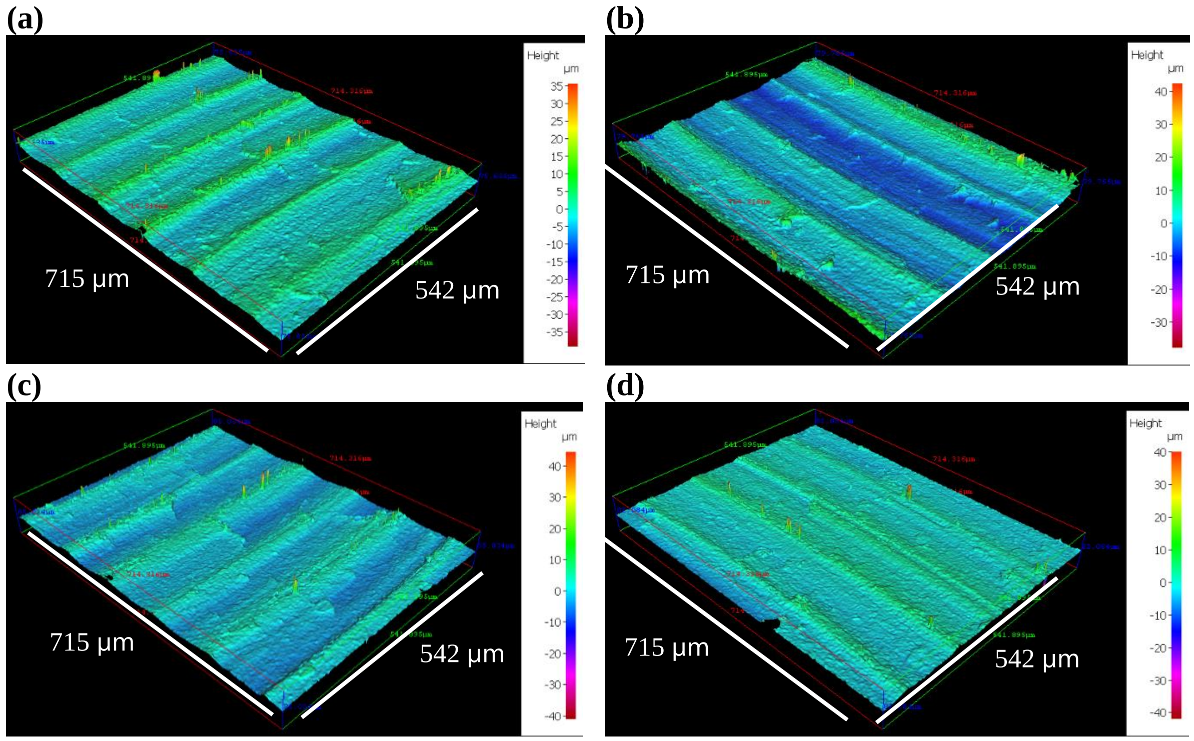

Figure 4. Three-dimensional optical surface profilometry of the cleaned UNS G10180 surfaces at day 28. AR coupons for: **(a,b)** abiotic and **(c,d)** biotic conditions, prior to exposure to anaerobic produced water media for 28 days.

Table 5. Quantitative surface roughness profiles for AR coupon samples on day 0 and day 28. R_a average roughness of profile, R_t maximum peak to valley height of roughness, R_z mean peak to valley height, R_p maximum peak height, R_v maximum valley height, R_c mean height of profile irregularities, R_{sm} mean spacing of profile irregularities, R_t/R_z extreme scratch/peak value of roughness profile (higher values (>=1) represent larger scratches/peaks).

Day	Reactor	R_a (µm)	R_t (µm)	R_z (µm)	R_p (µm)	R_v (µm)	R_c (µm)	R_{sm} (µm)	R_t/R_z (µm)
0	Abiotic	1.2±0.6	9.5±2.7	7.1±2.3	5.9±1.6	3.6±1.3	5.4±2.1	136±38	1.3±0.2
	Biotic	1.1±0.5	8.2±2.6	6.3±2.1	5.0±1.6	3.3±1.2	4.6±2.0	115±23	1.3±0.2
28	Abiotic	1.3±0.6	10.3±3.6	7.6±2.5	5.8±2.3	4.5±1.7	5.4±2.1	104±26	1.3±0.2
	Biotic	1.3±0.6	10.0±3.5	7.3±2.6	6.0±1.8	4.0±2.0	5.3±2.2	112±36	1.4±0.2

Table 6a. Quantitative SEM-EDS data collected from elemental mapping of the abiotic UNS G10180 carbon steel surfaces, after exposure to anaerobic produced water media for 28 days.

Statistic	O	F	Na	Mg	Al	Si	P	S	Cl	K	Ca	Mn	Fe	Ni	Cu	Mo	Rh	Sn	Ba	W
**Max**	37.35	8.94	22.92	1.30	0.00	4.49	1.43	2.33	23.16	0.82	9.63	0.00	46.00	0.00	0.00	0.00	2.33	0.00	7.65	1.74
**Min**	20.93	8.94	3.84	0.26	0.00	1.09	0.41	0.44	0.85	0.28	1.82	0.00	22.74	0.00	0.00	0.00	1.29	0.00	1.45	1.74
**Average**	29.88	8.94	11.29	0.80	-	3.38	0.70	1.42	8.03	0.50	5.69	-	33.89	-	-	-	1.74	-	3.87	1.74
**StDev**	3.56	-	4.66	0.20	-	0.82	0.22	0.48	6.13	0.18	1.76	-	7.03	-	-	-	0.39	-	1.26	-

Table 6b. Quantitative SEM-EDS data collected from elemental mapping of the biotic UNS G10180 carbon steel surfaces, after exposure to anaerobic produced water media for 28 days.

Statistic	O	F	Na	Mg	Al	Si	P	S	Cl	K	Ca	Mn	Fe	Ni	Cu	Mo	Rh	Sn	Ba	W
**Max**	50.17	0.00	40.61	0.64	0.27	2.99	0.00	1.01	13.63	0.24	1.85	4.72	82.72	0.96	1.23	0.57	0.00	0.93	0.00	0.00
**Min**	12.10	0.00	1.34	0.27	0.27	0.19	0.00	0.22	0.23	0.24	0.25	0.53	9.22	0.34	0.26	0.57	0.00	0.93	0.00	0.00
**Average**	25.12	-	6.51	0.43	0.27	0.59	-	0.57	2.58	0.24	0.55	1.24	65.24	0.65	0.79	0.57	-	0.93	-	-
**StDev**	6.53	-	7.12	0.12	-	0.64	-	0.26	3.22	-	0.38	0.86	11.06	0.44	0.35	-	-	-	-	-

Figure 7. Equivalent circuit model used to generate EIS parameters shown in Table S7.

① R1+(Q1/Q2+R2)

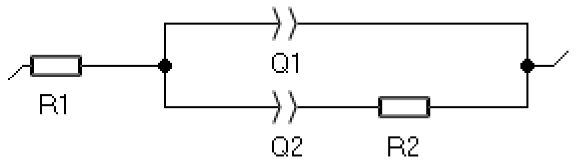

② R1+(Q1+R2)

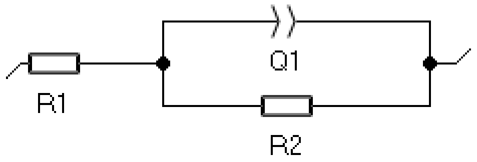

③ R1+Q1/(R2+Q2/R3)

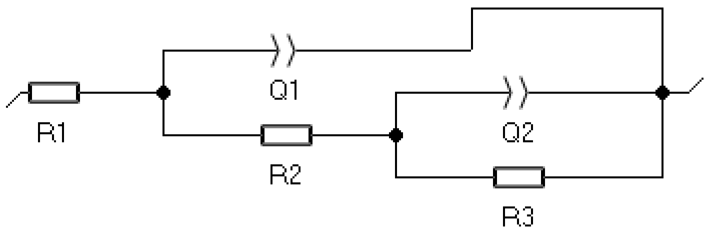

④ R1+Q1+(Q2/R3)

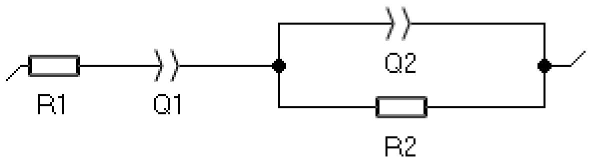

Table 8a. EIS parameters of the carbon steel coupons immersed in anaerobic produced water media for the abiotic condition on days 1, 7, 14, 21 and 28. **Equivalent circuit:** ① – $R_s+(Q_1/Q_2+R_{ct})$; ③ – $R_s+Q_1/(R_{film}+Q_2/R_{ct})$.

Day	Coupon	R_s / Ω cm^2	Q_1 / mΩ^{-1} cm^{-2} s^n	n_1	R_{film} / Ω cm^2	Q_2 / mΩ^{-1} cm^{-2} s^n	n_2	R_{ct} / Ω cm^2	χ^2 ×10^{-4}
1	AR ①	5.39±0.36	0.29±0.06	0.82±0.02	–	0.50±0.42	0.44±0.06	2921±2317	49
7	AR ①	4.90±0.40	0.72±0.06	0.84±0.03	–	0.43±0.19	0.68±0.17	1234±828	52
14	AR ①	4.32±2.16	2.01±1.09	0.75±0.09	–	1.34±1.06	0.72±0.14	382±559	42
21	AR ①	6.09±1.48	1.39±1.16	0.66±0.19	–	2.25±1.61	0.66±0.15	237±358	11
28	AR ③	5.37±3.41	0.55±0.58	0.64±0.22	2422±1385	0.81±0.51	0.63±0.23	176±304	29

Table 8b. EIS parameters of the carbon steel coupons immersed in anaerobic produced water media for the biotic condition on days 1, 7, 14, 21 and 28. **Equivalent circuit:** ① – $R_s+(Q_1/Q_2+R_{ct})$; ④– $R_s+Q_1+(Q_2/R_{ct})$.

Day	Coupon	R_s / Ω cm^2	Q_1 / mΩ^{-1} cm^{-2} s^n	n_1	Q_2 / mΩ^{-1} cm^{-2} s^n	n_2	R_{ct} / Ω cm^2	χ^2 ×10^{-4}
1	AR ①	5.22±0.38	0.38±0.15	0.86±0.02	1.42±0.72	0.38±0.11	419±322	34
7	AR ①	4.04±2.05	0.93±0.44	0.76±0.21	1.13±0.58	0.66±0.13	348±273	54
14	AR ④	4.74±0.59	3.20±1.33	0.79±0.02	2.52±1.31	0.89±0.06	42±20	50
21	AR ④	4.68±0.59	5.21±2.63	0.77±0.01	2.68±0.84	0.84±0.06	46±16	51
28	AR ④	4.74±0.36	6.97±2.97	0.81±0.08	4.73±0.76	0.82±0.03	85±107	30

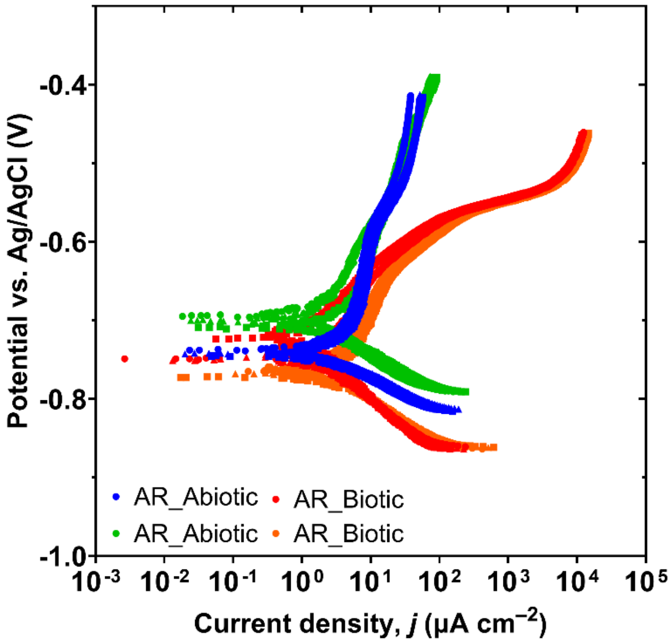

Figure 9. Potentiodynamic polarisation curves for the abiotic and biotic AR, UNS G10180 carbon steel coupons, at ambient temperature after exposure to anaerobic produced water media for 28 days. Scan rate of 0.5 mV s^{-1} and reactor stirrer at 50 rpm.

Table 10. Fitted electrochemical parameters from polarisation curves; comparison between the abiotic and biotic AR, UNS G10180 carbon steel coupons, after exposure to anaerobic produced water media for 28 days.

	Coupons	j_{corr} / µA cm^{-2}	E_{corr} *vs.* Ag/AgCl / V	β_a (mV dec^{-1})	β_c (mV dec^{-1})
**Abiotic**	AR	3.1 ± 0.8	-723 ± 23	240 ± 36	54 ± 7
**Biotic**	AR	0.7 ± 0.2	-748 ± 16	64 ± 2	51 ± 15

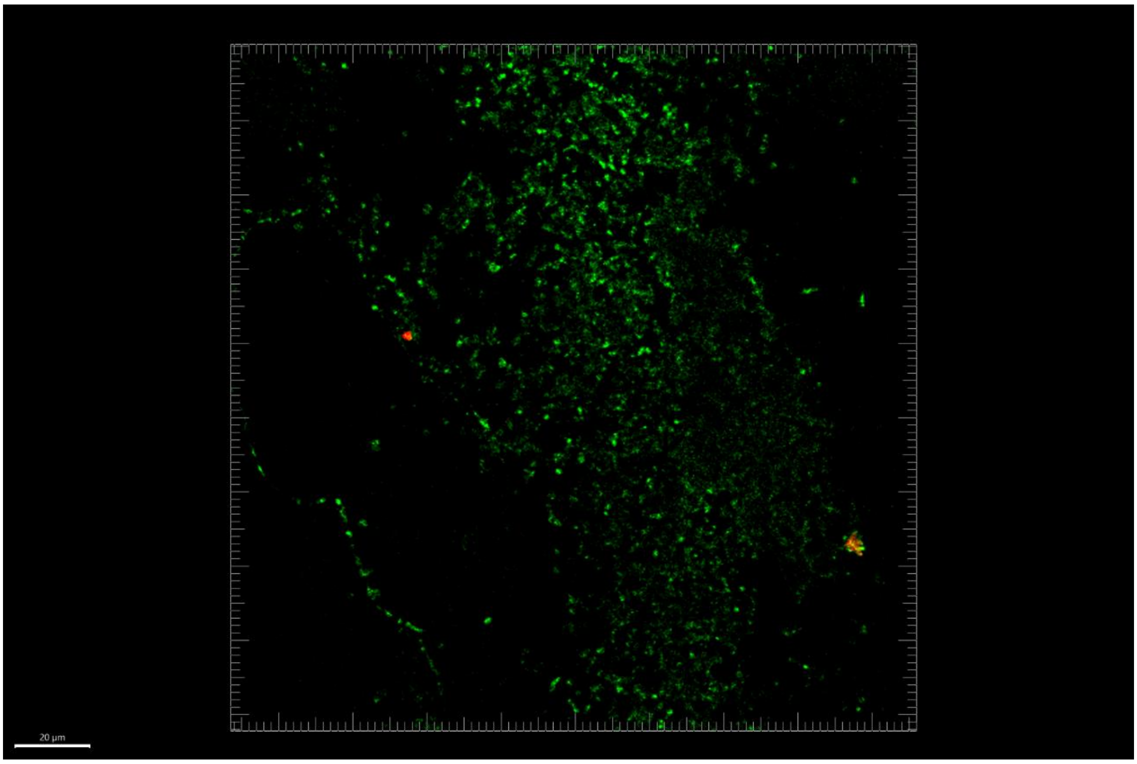

Figure 11. Confocal image of biofilm formed over UNS G10180 carbon steel surfaces for AR biofilms, after exposure to anaerobic produced water media for 28 days.

Table 12. List of top 25 microbial genera identified through 16S rRNA amplicon sequencing with two target region, V3-4 for bacteria and archaea, after exposure to anaerobic produced water media for 28 days.

Name	Sediment	Day0	Day28	AR
Marinobacterium	0.006341	0.001318	9.109	42.43
Vibrio	0.09423	32.41	0.1822	1.277
Marinobacter	0.003875	0.001538	30.26	2.837
Pseudomonas	0.273	0.005711	25.73	19.9
Halomonas	0.01233	0.000659	22.91	5.648
Sulfurovum	13.84	0.0246	0.0005165	0.0008302
Methanococcoides	0.03188	12.47	0.002238	NA
Malaciobacter	0.001585	0.0008786	1.755	9.184
Desulfosarcina	6.477	0.009885	NA	NA
Apibacter	0.02501	6.249	0.08867	0.2828
Methanococcus	0.1828	5.618	NA	NA
Desulfuromonas	5.464	0.01098	0.0001722	NA
Pseudodesulfovibrio	4.922	0.01955	0.002583	0.1962
Thiohalobacter	4.505	0.007029	NA	0.0002767
Idiomarina	0.0347	0.0004393	4.063	1.072
Geosporobacter	0.004051	3.942	0.02462	0.2779
Anaerotignum	0.001585	3.91	0.002755	0.01743
Fusobacterium	0.001761	3.861	0.002583	0.0005535
Flavobacterium	0.1402	3.579	0.1104	1.631
Kineobactrum	3.376	0.004174	0.0001722	NA
Hungatella	0.0001761	3.152	0.005165	0.03321
Wenzhouxiangella	3.111	0.003515	NA	0.0005535
Shewanella	0.02255	1.266	0.4752	3.07
Candidatus Prometheoarchaeum	2.843	0.03624	0.0003444	NA
Carnobacterium	0.0001761	NA	0.2726	2.626

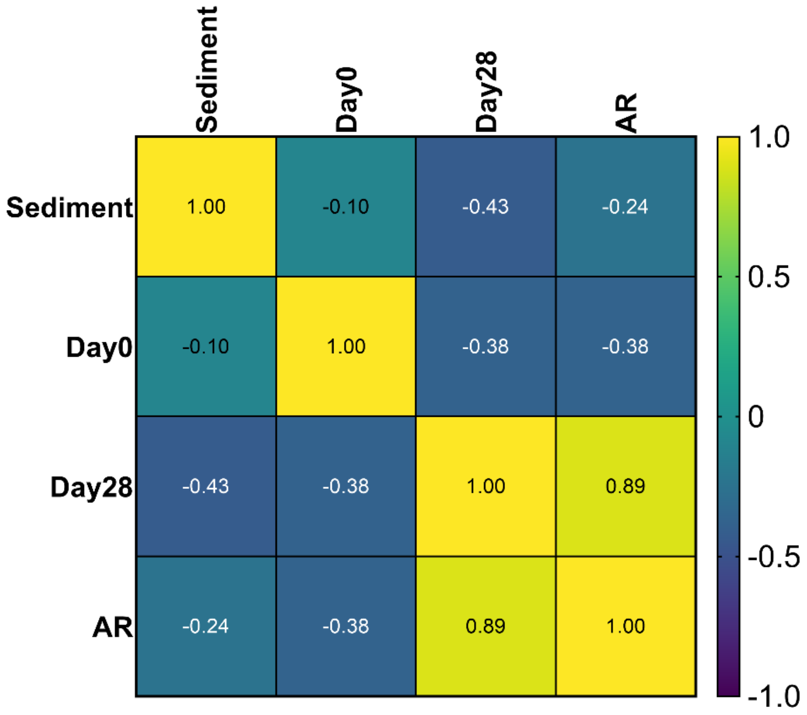

Figure 13. Spearman correlation coefficients for environmental marine sediment, Day 0, and Day 28 planktonic samples, and AR biofilms, after exposure to anaerobic produced water media for 28 days.

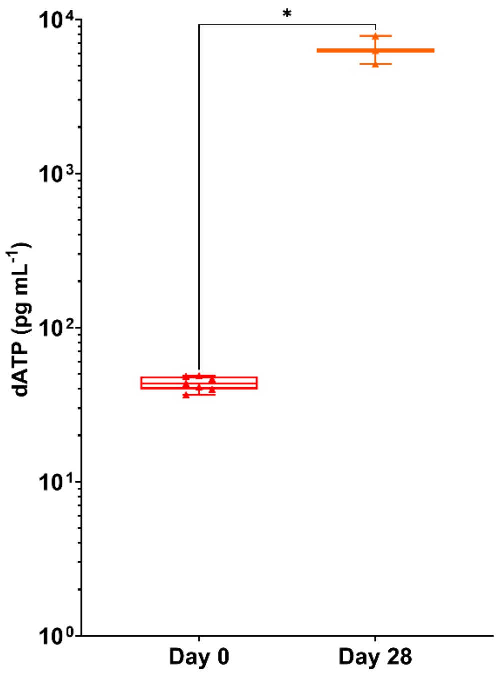

Figure 14. Dissolved ATP (dATP) concentrations comparing the anaerobic produced water media, taken on Day 0 and Day 28 ($P < 0.05$).
